# Cell deformability drives fluid-to-fluid phase transition in active cell monolayers

**DOI:** 10.1126/sciadv.adi8433

**Published:** 2024-05-08

**Authors:** Nen Saito, Shuji Ishihara

**Affiliations:** ^1^ Graduate School of Integrated Sciences for Life, Hiroshima University, Japan.; ^2^ Exploratory Research Center on Life and Living Systems, National Institutes of Natural Sciences, Japan.; ^3^ Universal Biology Institute, The University of Tokyo, Japan.; ^4^ Graduate School of Arts and Sciences, The University of Tokyo, Japan.

## Abstract

Cell deformability is an essential determinant for tissue-scale mechanical nature, such as fluidity and rigidity, and is thus crucial for tissue homeostasis and stable developmental processes. However, large-scale simulations of deformable cells have been restricted to those of polygonal-shaped cells, limiting our understanding of populations of arbitrarily deformable cells, such as mesenchymal, amoeboid cells, and nonconfluent epithelial cells. Here, we present an efficient approach for simulating large populations of nonpolygonally deformable cells with considerably higher computational efficiency than existing methods. Using the method, we demonstrate that the densely packed active cell population interacting via excluded volume interactions exhibits a fluid-to-fluid transition. An experimentally measurable index of topological defects, defined using the number of neighboring cells, is also proposed to characterize this transition. This study provides a flexible approach to tissue-scale cell population and a broader perspective on the biological fluid phases.

## Teaser

An efficient approach for simulating deformable cells reveals a fluid-to-fluid transition in monolayer cell populations.

## INTRODUCTION

In dense biological tissues, cells push against each other through mechanical interactions, causing collective cell migration and tissue deformation in concert with cell motility (*1*, *2*). The shapes of individual cells are not constant but can show drastic alternation as a result of the mechanical interaction and are, therefore, spatiotemporally heterogeneous. Such cell deformability facilitates cell rearrangements and is critical to tissue-scale rheological characteristics, including tissue elasticity and viscosity (*2*). Since such rheological characteristics are intimately involved in pivotal biological processes such as wound healing, embryogenesis, and cancer invasion, the exploration of emergent tissue-scale phenomena arising from the cell deformability has elicited considerable discussion and interest in recent years (*3*–*10*).

Epithelial cells forming a confluent monolayer are well described by the cell vertex model (VM), in which the cells are densely packed with no gaps, and their shapes are approximated by convex polygons (*11*, *12*). The VM was recently used to explain the experimentally observed rigidity transition (*5*, *6*) from a glassy phase, where the movements of cells are nearly frozen, to a fluidic phase, where frequent cell rearrangements result in tissue-scale collective motion. Despite the constant cell density with a volume fraction of one, the VM undergoes a rigidity transition, where the deformability of cells triggers a cascade of cell rearrangements. The transition occurs when the target cell shape index *q* defined by $\left(\text{cell perimeter}\right)/\sqrt{\left(\text{cell area}\right)}$ exceeds *q* = 3.81. The self-propelled Voronoi model that is almost equivalent to the VM, including the self-propelled force (*7*), also showed the same rigidity transition at <*q*> = 3.81, where <*q*> is the average value of the shape index over cells. These studies suggest that the rigidity transition is conditioned by the geometric parameter *q*, which is also supported experimentally (*8*). In addition, the VM was also suggested to exhibit the solid-solid transition due to the geometric frustration even without T1 transition (*13*).

On the other hand, it is yet to be well understood how highly deformable cells, such as nonconfluent epithelial cells and mesenchymal cells, for which polygonal approximation is inappropriate, behave at high density. Cells that deviate from a polygonal form (i.e., a shape that can be approximated by a convex polygon as seen in the VM) are observed ubiquitously across various biological processes, including development, regeneration, and pathogenesis, and it is important to clarify how a population of these cells acquire fluidity and mobility and how the emergent transitions differ from those in confluent epithelial cells (*6*, *7*). To simulate cells with nonpolygonal shapes, the phase-field model with self-propulsion was used to explore cells at high density (*14*), and it was shown that a rigidity transition similar to the VM occurs merely by considering the excluded volume interaction. A model of foam-like deformable cells with cell adhesion was also investigated (*9*), where strong cell adhesion mediates the rigidity transition. For both studies, the rigidity transition occurred at a similar <*q*> value in the VM. While the vertex and Voronoi models are subjected to a pronounced constraint, wherein cells share cell edges with their neighbors and exhibit transitions stemming from the geometric frustrations (*6*, *7*, *13*, *15*), such geometric constraints are much less severe in the phase-field model or the active particle. It is thus important to understand how this difference modifies the nature of the phase transition. However, the large-scale simulation of highly deformable cells is still challenging, and these models can only handle a smaller number of cells than the VM, and it remains to be elucidated whether a specific phase exists in nonpolygonal cells.

Here, we propose the Fourier contour cell model that allows us to handle up to 10^4^ deformable cells on a single CPU. The cells in the model have a round shape when isolated, whereas, in a high-density situation, the model can describe drastic cell deformation caused by the balance between the cell surface tension and excluded volume interactions. We demonstrate that a soft fluid phase specific to mesenchyme-like cells appears through excluded volume interactions and self-propulsion of each cell. The observed phase transitions were accompanied by the percolation of topological defects, providing a fresh perspective on mesenchymal cell dynamics that is experimentally verifiable.

## RESULTS

### Model

**Fig. 1.**
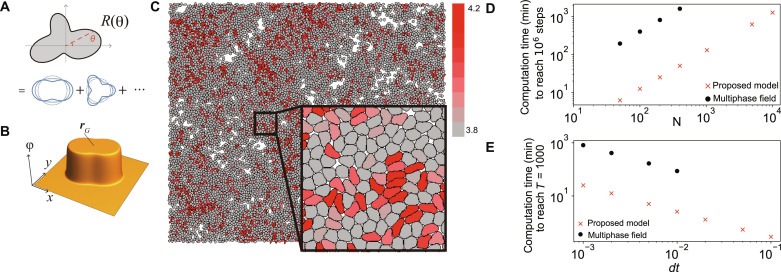
The schematic illustrations and the performance of the proposed model. (**A**) The polar coordinate representation of the cell contour is decomposed by the Fourier expansion into the independent Fourier modes. (**B**) The field representation of the cell by ϕ in which ϕ takes ϕ = 1 inside and ϕ = 0 outside of the cell. (**C**) A snapshot of the simulation with 10^4^ particles with volume density 0.8. Total simulation time steps are 5 × 10^6^. Parameters *v*_0_ = 0.06 and η = 0.01 are used. The red color represents the shape index $\left(\text{perimeter}/\sqrt{\text{area}}\right)$ for each particle. (**D** and **E**) Comparison of computational performance between the proposed model and the phase-field model. (D) The computation time (minutes) to reach 10^6^ simulation steps with *dt* = 10^−3^ for both models. (E) The computation time (minutes) to reach *T* = 1000 with a different *dt* for the number of cells *N* = 200. The phase-field method becomes unstable and shows divergence at *dt* > 0.01. These comparisons were performed using a single CPU (Intel Xeon Gold) and the same compiler (ICC compiler). For the proposed model, *v*_0_ = 0.04 and η = 0.01 were used. Detailed implementation and parameter sets for the multicellular phase-field model were given in Supplementary Text.

Deformable cells interacting in two-dimensional space are considered here. We propose the Fourier contour cell model in which the cell contour is expressed by polar coordinates *R*(θ) (Fig. 1A). The cell contour *R*(θ) for *i*th cell centered at the origin is expressed by a Fourier expansion up to *M*th order, as follows

$$R^{i}\left(\theta \right)=R_{0}\sum_{n=0}^{M}\;\left[a_{n}^{i}\text{cos}n\left(\theta -\theta^{i}\right)+b_{n}^{i}\text{sin}n\left(\theta -\theta^{i}\right)\right]$$ (1)

where $\left\{a_{n}^{i}\right\}$ and $\left\{b_{n}^{i}\right\}$ are Fourier coefficients, *R*_0_ is a constant parameter with dimension of length, and θ*^i^* denotes the orientation of the *i*th cell, which corresponds to the self-propulsion direction of the cell. In this study, we adopt *M* = 6. The effect of each mode on the cell shape is visualized in fig. S1. Considering the constraints that the cell area is constant at $\pi R_{0}^{2}$ and that the origin of the polar coordinates coincides with the cell center (i.e., the centroid), $a_{0}^{i}$, $a_{1}^{i}$, and $b_{1}^{i}$ are determined as $a_{0}^{i}=\sqrt{1-\sum_{n=1}^{M}\;\left[{\left(a_{n}^{i}\right)}^{2}+{\left(b_{n}^{i}\right)}^{2}\right]/2}$ and $a_{1}^{i}=b_{1}^{i}=0$, respectively. Furthermore, we impose a constraint $\sum_{n=1}^{M}\;\sqrt{{\left(a_{n}^{i}\right)}^{2}+{\left(b_{n}^{i}\right)}^{2}}<\sqrt{2/3}$ to avoid self-crossing of the contour (see the Supplementary Materials). The variables for describing the *i*th cell are composed of the cell center position $r_{c}^{i}$, orientation of the self-propulsion θ*^i^*, and Fourier coefficients to describe shapes $\left\{a_{n}^{i}\right\}$ and $\left\{b_{n}^{i}\right\}$ (*n* = 2, 3, …, *M*).

As the simplest interaction, only the excluded volume effect is considered. The interaction Hamiltonian $H_{\mathrm{int}}$ between *i*th and *j*th cells is given as an energetic penalty against the overlapped area. To compute this overlap, the field representation of the cell, originally used to represent the elliptical shape of the cell (*16*), was applied to describe deformable cells. The interior region of *i*th cell is described by ϕ*^i^*(**r**) ∼ 1 (Fig. 1B) using ϕ*^i^*(**r**) calculated as follows

$$\phi^{i}\left(r\right)=\frac{1}{2}+\frac{1}{2}\text{tanh}\{\frac{R^{i}[\theta \left(r,r_{c}^{i}\right)]-\Delta^{i}\left(r,r_{c}^{i}\right)}{\epsilon /2}\}$$ (2)

where $\theta \left(r,r_{c}^{i}\right)$ is the angle between $r-r_{c}^{i}$ and *x* axis, and $\Delta^{i}\left(r,r_{c}^{i}\right)=|r-r_{c}^{i}|$. With small ϵ, function ϕ*^i^*(**r**) sharply rises to 1 inside the cell (i.e., Δ*^i^* < *R^i^*) and drops to 0 outside the cell (Fig. 1B). Interface width between ϕ*^i^* = 0 and ϕ*^i^* = 1 domains is controlled by ϵ, which is considered infinitesimal below. The interaction Hamiltonian is then given by $H_{\text{int}}=\sum_{i<j}\;H_{\text{int}}^{\mathrm{ij}}=\sum_{i<j}\int \mathrm{dr}\phi^{i}\phi^{j}$, where ∑_*i*<*j*_ denotes summation for all *i*-*j* pairs, and we set the coefficient for the interaction to unity. We also incorporate the energy for penalizing interface length associated with the membrane tension $H_{l}=\sum_{i}\;H_{l}^{i}=\sum_{i}\;\eta \int_{-\pi }^{\pi }\;\sqrt{{\left(R^{i}\right)}^{2}+{\left(R^{\prime i}\right)}^{2}}d\theta$, where η is the tension parameter, and *R*′^*i*^ denotes the derivative of *R^i^* with respect to θ. This term does not depend on **r***i_c_* and θ*^i^*. We then obtain the total Hamiltonian $H=H_{\mathrm{int}}+H_{i}$.

The time-evolution equation for $r_{c}^{i}$ is derived by $-dH/dr_{c}^{i}=-dH_{\text{int}}/dr_{c}^{i}$ and the self-propulsion term **v***^i^* = (*v*_0_ cosθ*^i^*, *v*_0_ sinθ*^i^*), which is often introduced for representing active motion of cells (*16*–*23*)

$${\dot{r}}_{c}^{i}=v^{i}-\mu_{r}\frac{dH}{dr_{c}^{i}}=v^{i}+\mu_{r}\int dr\frac{\delta H}{{\delta \phi }^{i}}\nabla \phi^{i}=v^{i}+\mu_{r}\sum_{j\neq i}\int \mathrm{dr}\phi^{j}|\nabla \phi^{i}|\frac{\nabla \phi^{i}}{|\nabla \phi^{i}|}$$ (3)

where μ*_r_* is the mobility parameter for $r_{c}^{i}$. By taking the sharp interface limit ϵ → 0, ∣∇ϕ*^i^*∣ becomes the surface delta function δ*_s_*(|**r** − **r***^i^_c_*| − *R^i^*) (see Supplementary Text), and the area integral is replaced by an integral along the cell contour ∑_*j*≠*i*_ ∮ *dsϕ^j^***n***^i^* where **n***^i^* denotes the normal vector of the contour. By using **e**_θ_ = (cosθ, sinθ) and **e**_⊥_ = (−sinθ, cosθ), ${\dot{r}}_{c}^{i}$ is given by

$${\dot{r}}_{c}^{i}=v^{i}+\mu_{r}\sum_{j\neq i}\;\int_{0}^{2\pi }\;d{\theta \phi }^{j}\left[{R\prime }^{i}\left(\theta \right)e_{⊥}-R^{i}\left(\theta \right)e_{\theta }\right]$$ (4)

At the integral, ϕ*^j^* = 1 when the position $r_{c}^{i}+R^{i}e_{\theta }$ on the contour in the θ-direction of the *i*th cell is occupied by the *j*th cell, and ϕ*^j^* = 0 otherwise. Likewise, the time evolution of θ*^i^* is obtained as ${\dot{\theta }}^{i}=-\mu_{\theta }dH/d\theta^{i}+\sqrt{2D_{r}}\xi^{i}$, where the first term in the right-hand side represents the torque term caused by the cell collisions (see the Supplementary Materials), and the second term denotes a noise term. For simplicity, we incorporate the noise $\sqrt{2D_{r}}\xi^{i}$, where ξ*^i^* represents a normalized white Gaussian noise, only into the equation of ${\dot{\theta }}^{i}$. This formulation indicates that the cell does not change its intrinsic cell polarity direction (i.e., self-propulsion direction) except for the noise effect, but the cell itself can rotate due to the torque produced by the collision, and the polarity direction also rotates in accordance with the rotation of the cell. The equations for {*a^i^_n_*} and {*b^i^_n_*} (*n* = 2, 3, …, *M*) are also obtained by ${\dot{a}}_{n}^{i}\propto -dH/{\mathrm{da}}_{n}^{i}$ and ${\dot{b}}_{n}^{i}\propto -dH/{\mathrm{db}}_{n}^{i}$. Note that the tension term in the Hamiltonian affects only the equations for {*a_n_*} and {*b_n_*}. The numerical integration along the cell contour θ was performed by discretizing 0 ≤ θ ≤ 2π by 40 points, whereas the integration with respect to time *t* was performed using the Euler-Maruyama method with *dt* = 5 × 10^−3^. The dynamics in the cell center $r_{c}^{i}$, orientation θ*^i^*, and cell shape $\left\{a_{n}^{i},b_{n}^{i}\right\}$ are described by a set of ordinary differential equations, where the two-dimensional numerical integration in Eq. 3 is reduced to a one-dimensional integration. This considerably reduces the computation time. Figure 1C shows a representative simulation snapshot of *N* = 10^4^ active deformable particles (see also movie S1 in the Supplementary Materials), which may not be feasible using the phase-field method (*14*, *24*, *25*).

We also quantified the computational performance of the proposed model compared with the phase-field model (Fig. 1, D and E). Figure 1D shows the computation time to reach 10^6^ simulation steps with *dt* = 10^−3^ for both the proposed model and the multiphase-field model using a single CPU, which demonstrates that the proposed method is more than 30 times faster in the calculation per single time step. Figure 1E shows the computation time to reach *T* = 1000 with a different *dt*. The computation by the phase-field method became unstable at *dt* > 0.01, whereas the proposed model showed no noticeable instabilities until around *dt* = 0.05 or 0.1. These estimates indicate the superiority of the proposed model, which achieves computation speeds up to 150 to 300 times faster than the phase-field model. Note, however, that this comparison was naive in that it compared models with different detailed assumptions, and further validation is needed in the future.

### Active deformable cells undergo cell shape transition

Using the proposed model, we study active deformable particles densely packed with volume fraction ρ = 0.95 and interact with each other solely through excluded volume interactions. The simulation box size was set as $L_{x}:L_{y}=2:\sqrt{3}$, and the particles were initially positioned on a perfect hexagonal lattice with random θ*^i^*. Although up to *N* = 10^4^ particles were handled, calculations were performed mainly with *N* = 1024 when searching the parameter space.

**Fig. 2.**
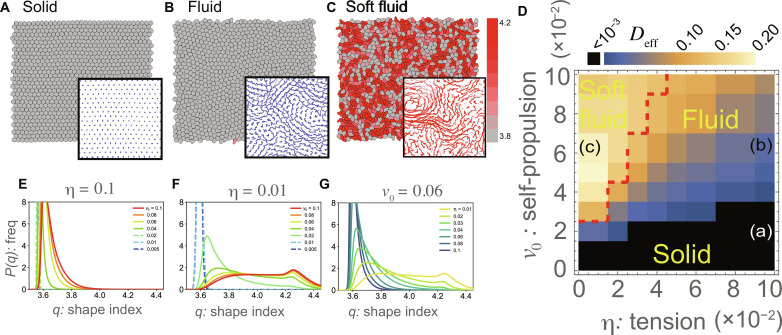
Phases for the proposed model. (**A** to **C**) The snapshot of the simulation for the solid phase (A), the fluid phase (B), and the soft fluid phase (C). For cells with the shape index *q* > 3.81, the value of *q* is indicated by the color scale from gray to red. The insets denote particle trajectories within 10^5^ steps, and the color scale from blue to red represents the average of *q* during the time steps. (**D**) Phase diagram of the model against the tension η versus the self-propulsion velocity *v*_0_. The color indicates the effective diffusion constant *D*_eff_ evaluated from simulation with *N* = 1024 and 8 × 10^7^ time steps. The red dashed line represents 〈*q*〉 = 3.81. A dimensionless version of this phase diagram is shown in fig. S5. (**E** to **G**) Distributions of the shape index for η = 0.1 (E) and η = 0.01 (F) with varying *v*_0_ and that for *v*_0_ = 0.06 with varying η (G). Thick lines indicate the parameters for the fluid or soft fluid phase, and the dashed lines represent the parameters for the solid phase.

Representative snapshots are shown in Fig. 2 (A to C). At a small self-propulsion *v*_0_ and a large tension η, the system exhibits a solid phase (Fig. 2A), where the particles form a hexagonal lattice and hardly show rearrangements (particle trajectories are shown in the inset of Fig. 2A; see also movie S2 in the Supplementary Materials). Accordingly, the mean square displacement (MSD) measured from the trajectories of the cell centers exhibits a saturation curve (fig. S2; η = 0.08 and 0.1). The particles in this phase were nearly circular without a large deformation (Fig. 2A). At large *v*_0_ and large η, the system is in the fluid phase (Fig. 2B), where the particle positions are frequently rearranged (Fig. 2B inset; see also movie S3 in the Supplementary Materials) and the MSD curve exhibited linear growth, indicating diffusive dynamics of cells (fig. S2; η = 0.02 to 0.06). The particle shapes remained almost circular (Fig. 2B; the degree of deformation is denoted by red color). At a small η and large *v*_0_, where the particles are easily deformed, the third phase, which we call the soft fluid phase, emerges (Fig. 2C; movie S4 in the Supplementary Materials). In this phase, largely deformed particles and relatively circular particles coexist, and they exhibit fluidic characteristics, such as frequent rearrangements (Fig. 2C, inset) and a linearly increasing MSD curve (fig. S2; η = 0.01).

Figure 2D illustrates the phase diagrams for η and *v*_0_ (see also fig. S5). Color represents the effective diffusion coefficient *D*_eff_ = *D*_s_/*D*_0_, where $D_{0}=v_{0}^{2}/2D_{r}$ is the diffusion coefficient for an isolated cell. and $D_{s}=\underset{t\to \infty }{\text{lim}}\left(\langle {\left(\Delta r\right)}^{2}\rangle -{\langle \Delta r\rangle }^{2}\right)/4t$ were estimated from the trajectories of the cell centers. The solid phase is determined by the criterion *D*_eff_ < 10^−3^, which is consistent with the MSD curves showing saturation (figs. S2 and S3). The solid-fluid phase boundary in Fig. 2D indicates that the rigidity-fluidity transition can occur not only by changing the self-propulsion velocity *v*_0_ but also by changing the cell deformability η. For *v*_0_ = 0.03, *D*_eff_ varies abruptly at the phase boundary (between η = 0.06 and 0.08), and the MSD curves (fig. S2) change from a diffusion line (η ≤ 0.06) to a saturating curve (η ≥ 0.08). The phase boundary is also associated with a decrease in the hexatic order parameter ∣Ψ_6_∣ (fig. S4) defined by $\Psi_{6}\left(r\right)=<\sum_{j=1}^{n_{i}}\;e^{i6\theta_{\mathrm{ij}}}/n_{i}>$, where θ*_ij_* is the angle of the link connecting *i*th cell’s center to the adjacent *j*th cell’s center, *n_i_* is the number of adjacent pairs, and 〈〉 denotes the average over all cells.

### Fluid-fluid phase transition is characterized by the percolation of the topological defects

The phase boundary between the fluid and soft fluid is not straightforwardly determined. As these two fluid phases have distinct cell shape characteristics, the distribution of the shape index *q* is measured (Fig. 2, E to G) to characterize the phases. For a large η (high tension), the shape distribution displays a sharp, unique peak at *q* < 3.81 regardless of whether it is in the solid phase (small *v*_0_; Fig. 2E, dashed lines) or fluid phase (large *v*_0_; Fig. 2E, thick lines). On the other hand, for a small η (low tension), a sharply peaked distribution at *q* < 3.81 in the solid phase (small *v*_0_; Fig. 2F, dashed lines) turns into a long-tailed distribution as *v*_0_ increases and then into a bimodal distribution (Fig. 2F, thick lines). Changes in the distribution from unimodal to bimodal also occur without crossing the solid phase. For a fixed value of *v*_0_ = 0.06, as η decreases, we observe the appearance of a bimodal distribution with a second peak at *q* > 3.81 from the unimodal distribution with a peak at *q* < 3.81 (Fig. 2G). This bimodal distribution clearly illustrates the coexistence of largely deformed and relatively circular cells. Accordingly, the mean <*q*> increases around the region with a small η and high *v*_0_ (fig. S6). On the basis of this change in the distribution and the previously reported transition point in the VM (*6*, *7*), we tentatively defined the phase boundary as the mean shape index 〈*q*〉 = 3.81 (Fig. 2D, red dashed line). This heuristic definition will be justified below.

We also examined the effect of the Fourier mode cutoff *M* by performing the simulations with *M* = 12 (i.e., up to the 12th Fourier mode was computed) and confirmed that the qualitatively same results were obtained (fig. S7). With *M* = 12, cell shapes in the soft fluid phase (fig. S7C) are rounder than those with *M* = 6 (Fig. 2C), resulting in a slight shift of the second peak in the shape index distribution (fig. S7F). These results suggest that this change in the truncation does not qualitatively alter the obtained results (i.e., the presence of two fluid phases), but it can affect the roundness or elongation of the cell. Similarly, the qualitatively same results were obtained for the simulation with the different packing fraction 0.98 (fig. S8), that with a different number of discretized points on the contour (= 80) (fig. S9), that with a different mobility parameter μ*_ab_* (fig. S11), and that with a soft constraint on the area conservation (see the Supplementary Materials and fig. S10) instead of the hard area constraint adopted in the main text. Furthermore, we examined the validity of the intrinsic assumption that the cell shape is described by a star domain with a single-valued function *R*(θ). We evaluated the maximum value of ∣*dR^i^*/*d*θ∣ in each cell, because a large jump in *R^i^*(θ) is expected in the vicinity of the breakdown of the single-value assumption (see fig. S12A). The distribution in max_θ_∣*dR^i^*/*d*θ∣ (fig. S12B) shows that this value is at most about 2.0, much smaller than its theoretical upper bound ∼5.0, suggesting that the single-value assumption still holds in both soft fluid and fluid phases.

**Fig. 3.**
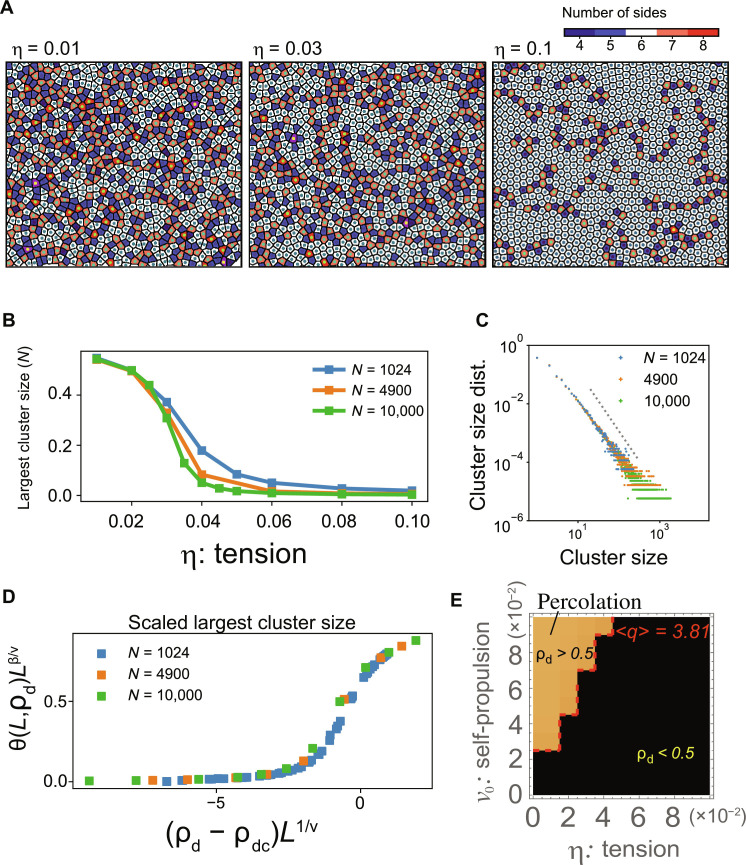
Percolation of the topological defects. (**A**) Snapshots of the Voronoi cells for η = 0.01 (left), 0.03 (middle), and 0.1 (right), calculated from Voronoi tessellation of the cell centers $\left\{r_{c}^{i}\right\}$. The colored cells represent topological defects with neighbor cell numbers other than 6. (**B**) The normalized largest cluster size plotted against η with *v*_0_ = 0.06 for *N* = 1024, 4900, and 10,000. (**C**) The cluster size distribution for *v*_0_ = 0.06 and η = 0.03. The dotted line denotes *y* ∝ *x*^−187/91^. (**D**) The scaled largest cluster size is plotted against the scaled defect ratio ρ_d_, where the percolation threshold ρ_d_ = 1/2 and the scaling exponents *v* = 4/3 and β = 5/36 are used. See also fig. S15 for the plot without the scaling. (**E**) The parameter region for the defect percolation is in good agreement with the line for 〈*q*〉 = 3.81.

To further examine the boundary between the fluid and soft fluid phases, we investigated behaviors of topological defects characterized by miscoordinated particles with respect to a perfect hexagonal lattice. After performing Voronoi tessellation using particle centers, $\left\{r_{c}^{i}\right\}$, Voronoi cells with other than six neighbors are defined as defect particles. Figure 3A shows the defect particles indicated by the colors for *v*_0_ = 0.06 at η = 0.01, 0.03, and 0.1. As η decreases from 0.1 to 0.01, the number of defect particles increases, and they are connected to form a large cluster (Fig. 3A). Figure 3B demonstrates a clear hallmark of the percolation transition; the largest cluster size of the connected defect particles exhibited a sharp transition between η = 0.02 and 0.06, and the transition becomes sharper with increasing system size *N* with a fixed density. Consistently, the size distribution of the connected clusters of the defect particles exhibited a power law near the critical point η = 0.03 for *v*_0_ = 0.06 (Fig. 3C). The power exponent is close to the critical exponent for two-dimensional percolation, 187/91 (*26*). The cluster size distributions for the other values of η are shown in fig. S13. In contrast to the largest cluster size, the defect ratio ρ_d_, defined as the average number of defect particles/*N*^2^, does not exhibit a drastic change, such as a transition. During a decrease in η, ρ_d_ increases smoothly, and its curve does not change with increasing *N* (fig. S14). The scatterplot of the defect ratio versus the size of the largest cluster for all examined parameters collapses to a master curve (Fig. 3D; see also fig. S15 for the unscaled version) by applying the percolation threshold ρ_dc_ = 0.5 for two-dimensional site percolation in the triangular lattice and its scaling exponents *v* = 4/3 and β = 5/36. This result clearly demonstrates the existence of percolation transition at ρ_dc_ = 0.5. The parameter region with ρ_d_ > 0.5 in the phase diagram coincided with the region with 〈*q*〉 > 3.81 (Fig. 3E; see also fig. S6), indicating that the percolation transition separates the soft fluid phase from the fluid phase.

### The translational and orientational correlations decay exponentially both in fluid and soft fluid phases

We also examined whether or not the fluid/soft fluid transition found in the proposed model has some relation to the hexatic/fluid phase transition in Kosterlitz-Thouless- Halperin-Nelson-Young (KTHNY) theory. According to the KTHNY theory (*27*, *28*), two-dimensional crystals display two-step melting. At the first transition, the quasi–long-range order of a crystal is destroyed by the dissociation of the thermally excited pairs of dislocations, which is a linear crystallographic defect, while the sixfold orientational order of the lattice is maintained. This phase is referred to as the hexatic phase. Subsequently, the hexatic phase undergoes the second phase transition to the isotropic fluid phase by losing the orientational order mediated by the dissociation of disclinations (*27*, *28*), a defect in which the rotational symmetry is broken. This two-step melting was confirmed in passive (*28*) and active discs (*29*), as well as in the Voronoi model (*30*, *31*). The question that arises here is whether the fluid and soft fluid phases in the proposed model correspond to either hexatic or isotropic fluid phases in the KTHNY theory, and whether the hexatic phase can be observed in this model. We addressed these issues by closely examining the transition from the solid to the fluid phase. We measured the pair translational correlation function *G_r_*(Δ*x*, 0) (*28*), which is calculated using the one-dimensional cut of the two-dimensional histogram *G_**r**_*(Δ**r**) of the pair distance **Δr** = **r_j_** − **r_i_** of an *i*-*j* pair of particle centers, and the orientational correlation function *G*_6_(∣**Δr**∣) defined by $G_{6}\left(|\Delta r|\right)=\langle \Psi_{6}\left(r_{i}\right)\cdot \Psi_{6}^{*}\left(r_{j}\right)\rangle$.

**Fig. 4.**
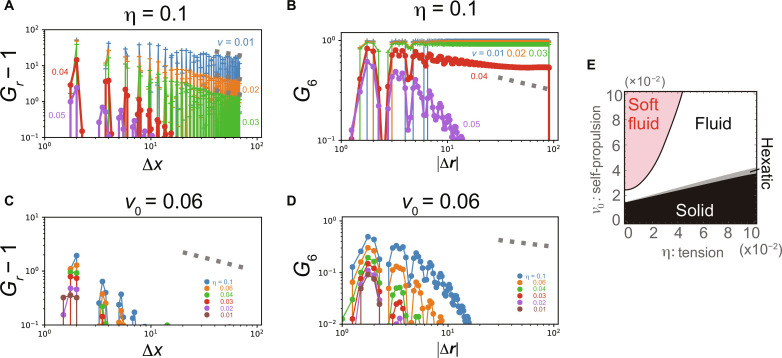
The translational and orientational correlation functions. (**A**) The translational correlation functions *G_r_*(Δ*x*, 0) − 1 for η = 0.1 are plotted from *v*_0_ = 0.01 (solid phase) to *v*_0_ = 0.05 (fluid phase). Gray dashed lines represent *G_r_* − 1 ∝ (Δ*x*)^−1/3^. (**B**) The orientational correlation functions *G*_6_(∣**Δr**∣) for η = 0.1 are plotted from *v*_0_ = 0.01 (solid phase) to *v*_0_ = 0.05 (fluid phase). Gray dashed lines represent *G*_6_ ∝ ∣**Δr**∣^−^^1/4^. (**C** and **D**) *G_r_*(Δ*x*, 0) − 1 (C) and *G*_6_(∣**Δr**∣) (D) for *v*_0_ = 0.06 are plotted from η = 0.1 (fluid phase) to η = 0.01 (soft fluid phase). Gray dashed lines represent *G_r_* − 1 ∝ (Δ*x*)^−1/3^ (C) and *G*_6_ ∝ ∣**Δr**∣^−^^1/4^ (D). All calculations in (A) to (D) are based on simulation with *N* = 4900 and 2 × 10^7^ steps. (**E**) Summarized phase diagram.

We calculated *G_r_*(Δ*x*, 0) and *G*_6_(∣**Δr**∣) by fixing η = 0.1 and increasing *v*_0_ from 0.01 to 0.05. As shown in Fig. 4A, from *v*_0_ = 0.01 to 0.03, the translational order *G_r_*(Δ*x*, 0) decays algebraically with exponent −1/3, corresponding to the solid phase in the KTHNY theory. *G_r_*(Δ*x*, 0) decreases exponentially for *v*_0_ from 0.04 to 0.05. The orientational order *G*_6_(∣**Δr**∣) shown in Fig. 4B decays more slowly than *G*_6_(∣**Δr**∣) ∝ ∣**Δr**∣^−^^1/4^ for *v*_0_ from 0.01 to 0.04, corresponding to solid or hexatic phases, and decreases faster than ∣**Δr**∣^−^^1/4^ for *v* = 0.04 and 0.05. These results indicate that the solid and fluid phases in the proposed model correspond to the solid and isotropic fluid phases in the KTHNY theory, and the hexatic phase would exist between them for *v*_0_ = 0.04 and η = 0.1 (Fig. 4, A and B). During the transition from the fluid phase to the soft fluid phase, *G_r_*(Δ*x*, 0) and *G*_6_(∣**Δr**∣) decayed exponentially (Fig. 4, C and D; *v*_0_ = 0.06 and η is changed), indicating that both translational and orientational orders are already broken. From these results, we conclude that both the fluid and soft fluid phases in the proposed model are classified into the isotropic fluid phase in terms of KTHNY theory and that the fluid/soft fluid transition is independent of transitions predicted in the KTHNY theory. The soft fluid phase is a phase that can be described by the percolation of topological defects but not by breaking orientational or translational orders. This scenario is summarized in the phase diagram shown in Fig. 4E.

## DISCUSSION

The present study proposes a numerical model of deformable cells based on the Fourier expansion of the cell contour, which provides a computationally efficient method for describing irregular cell shape deformations similar to mesenchymal and ameboid cells. Up to 10^4^ cells were handled using a single CPU, which may not be feasible in the phase-field method (*14*, *24*, *25*) or spring-beads models (*9*). Similar models based on the expansion of cell contours have also been proposed (*32*, *33*, *34*, *35*); however, these models had difficulty in simulating a tightly packed situation and torque effects, and thus, phenomena such as glassy dynamics and rigidity transition do not occur (*35*). Our approach is based on the field representation of cell shape ϕ*^i^*, which enables simple and efficient computation of the excluded volume effect at high density and allows us to incorporate the torque effect appropriately, which can alter collective behaviors (*16*, *36*).

The proposed model demonstrated that density-independent rigidity transition occurs by changing deformability η solely from the excluded volume interactions and self-propulsion. Although such a transition was reported in a previous study (*14*), we further elucidated the emergence of two types of fluid phases: the soft fluid phase, where the drastically deformed cells coexist with less deformed cells as is demonstrated in the bimodality in the shape index distribution, and the fluid phase, where individual cells remain almost circular with the unimodal shape index distribution. The soft fluid phase is similar to the fluid phase in the cell VM where cell shapes are drastically deformed with 〈*q*〉 > 3.81 (*6*, *7*) and the arrangement of the cell is far from regular. On the other hand, the fluid phase is akin to the fluidic phase in the active Brownian particle (ABP) model in which the cell shapes remain circular and the irregularity of the particle arrangement is relatively small. The model proposed in this study is capable of describing cell dynamics akin to both VM and ABP scenarios, thereby allowing the emergence of both of the fluid phases. This duality of the model could bring the transition between a fluid phase akin to the fluid in ABP to the soft fluid akin to the fluid in VM with 〈*q*〉 > 3.81, providing a possible interpretation for the fluid-to-fluid transition at 〈*q*〉 = 3.81. For the soft fluid phase, it would also be interesting to consider the various phenomena discussed in the extensions of VM, such as the existence of different routes to fluidization (*37*) and the presence of a gas-like phase (*38*).

The fluid/soft fluid transition is well-characterized by the percolation of topological defects. Recently, such defects percolation has been studied in equilibrium disks and ABP systems by changing the packing fraction, and it was found that percolation occurs around the hexatic-fluid phase transition boundary (*39*). This difference between the previous study and our study may be due to the density-dependent/independent nature of the transition or the absence/presence of deformability of cells. The percolation found in the present study also differs from those related to the rigidity transition, such as density-dependent percolation in embryogenesis (*10*) or stress field percolation around the solid-fluid transition in the epithelial monolayer (*40*). The defect percolation occurs at defect ratio = 0.5 (Fig. 3E). Given that defects are associated with largely deformed cells, the defect percolation occurs when more than half of the cells are highly deformed, causing fragmentation of the global connection of the locally ordered cell population (i.e., nondefect cells). In this sense, a possible scenario is that the defect percolation is caused by the cooperative flow of cell populations and the deformability of the cells.

The analysis of the translational and orientational order parameters in our model revealed that the fluid and soft fluid phases have the characteristics of an isotropic fluid, and the fluid/soft fluid transition has no relation to the hexatic/isotropic fluid transition in the KTHNY theory. In addition, the hexatic phase would exist around the solid/fluid transition point [see Fig. 4 (A and B; η = 0.04) and Fig. 4E], indicating that three fluid phases can potentially appear in the active deformable cell population. Considering the previous studies using the particle systems (*28*, *29*, *41*–*44*), the solid-hexatic and hexatic-fluid transitions would occur for any tension region (Fig. 4E). The hexatic phase in deformable particles differs markedly from that in particles of constant shape interacting via a soft repulsive potential (*28*, *29*, *41*–*44*). In the latter model, no shape deformation occurs, and the hexatic transition appears only through changes in packing fraction. This difference is critically important for the potential presence of the “hexatic” cell population in dense biological tissues.

The previous study (*14*) investigated soft particles by using the phase-field model and reported discontinuous-like transition when cellular tension is high enough. Compared to the density we addressed, they studied a much denser situation where the overlap between particles is much more severe, which may be the reason why the discontinuous-like transition was not observed in our study. Also with respect to the hexatic phase, the accurate determination of the hexatic phase generally requires a larger number of particles and longer relaxation times than in the present and previous phase-field studies. Further studies extended with parallel or GPU computations are needed to clarify the hexatic phase in deformable particles. In addition, there are other differences between the phase-field model (*14*) and ours in that (i) the present model only allows the star-shaped deformation with a finite number of Fourier modes and (ii) the existence of torque term (−dH/*d*θ*^i^* in *d*θ*^i^*/*dt*) in our model. The effect of these differences should be further investigated.

The fluidic collective motion of actual cells can be characterized using the present theoretical framework. The packing of cell populations with nonpolygonal shapes appears in embryo genesis (*9*) and in vitro experiments on cultured cells with suppressed cell-cell adhesion (*45*), and they potentially display fluidic collective motion by cell softening. The percolation of topological defects can be detected under these experimental conditions and provides insights into a possible fluid/fluid transition in the cell population.

The proposed model also has the flexibility to incorporate various ingredients, such as cell size differences, cell adhesion, and cell division, which need to be addressed elsewhere. The interaction Hamiltonian is based on the overlapped area between two cells, but its extension to a harder core potential is also possible. Although the present framework is limited to describing a star domain (radially convex set), where the contour can be described by the polar coordinate with a single-valued function *R^i^*(θ) and cannot be applied to an extreme deformation, such as ameboid cells typically show, an extension to use other orthogonal functions, such as the elliptic Fourier descriptors (*46*–*48*), would be possible and needs to be addressed. Extension to a three-dimensional model using spherical harmonic expansions instead of Fourier series expansions can also be considered. Thus, the proposed model, which enables a simple and efficient computation of deformable cells, can be a pivotal tool for studying cell populations.

## MATERIALS AND METHODS

### Model equation for the proposed model

Deformable cells interacting in two-dimensional space are considered. A cell contour is assumed to be represented by the polar coordinates using the single-valued function *R^i^*(θ) (Fig. 1A). The contour *R^i^*(θ) of the *i*th cell centered at $r_{c}^{i}$ is then expressed by a Fourier expansion up to *M*th order as follows

$$R^{i}\left(\theta \right)=R_{0}\{\sqrt{1-\sum_{n=2}^{M}\;\frac{{\left(a_{n}^{i}\right)}^{2}+{\left(b_{n}^{i}\right)}^{2}}{2}}+\sum_{n=2}^{M}\;[a_{n}^{i}\text{cos}n\left(\theta -\theta^{i}\right)+b_{n}^{i}\text{sin}n\left(\theta -\theta^{i}\right)]\}$$ (5)

The absence of the first-order coefficients $a_{1}^{i}$ and $b_{1}^{i}$ guarantees that the centroid of the cell is fixed at the origin of the expansion. The first term in the above equation also ensures the constant cell area $\pi R_{0}^{2}$. A set of variables for *i*th cell is $r_{c}^{i}$, θ*^i^*, $\left\{a_{n}^{i}\right\}$, and $\left\{b_{n}^{i}\right\}$. The time evolution equations for these variables are given as follows

$$\begin{array}{c} {\dot{r}}_{c}^{i}=v^{i}+\mu_{r}\sum_{j\neq i}\;\int_{-\pi }^{\pi }\;d{\theta \phi }^{j}[{R\prime }^{i}\left(\theta \right)e_{⊥}-R^{i}\left(\theta \right)e_{\theta }]\\ {\dot{\theta }}^{i}=\mu_{\theta }\sum_{j\neq i}\;\int_{-\pi }^{\pi }\;d{\theta \phi }^{j}{R\prime }^{i}\left(\theta \right)R^{i}\left(\theta \right)+\sqrt{2D_{r}}\xi^{i}\\ {\dot{a}}_{n}^{i}=-\mu_{\mathrm{ab}}\eta \int_{-\pi }^{\pi }\;R_{0}\frac{R^{i}[-\frac{a_{n}^{i}}{2\sqrt{1-\sum_{n}\;[{\left(a_{n}^{i}\right)}^{2}+{\left(b_{n}^{i}\right)}^{2}]/2}}+\text{cos}n\left(\theta -\theta^{i}\right)]-{R\prime }^{i}n\text{sin}n\left(\theta -\theta^{i}\right)}{\sqrt{{\left(R^{i}\right)}^{2}+{\left({R\prime }^{i}\right)}^{2}}}d\theta \\ -\mu_{\mathrm{ab}}\sum_{j\neq i}\;\int_{-\pi }^{\pi }\;d{\theta \phi }^{j}R^{i}\left(\theta \right)R_{0}[-\frac{a_{n}^{i}}{2\sqrt{1-\sum_{n}[\;{\left(a_{n}^{i}\right)}^{2}+{\left(b_{n}^{i}\right)}^{2}]/2}}+\text{cos}\text{n}\left(\theta -\theta^{i}\right)]\\ {\dot{b}}_{n}^{i}=-\mu_{\mathrm{ab}}\eta \int_{-\pi }^{\pi }\;R_{0}\frac{R^{i}[-\frac{b_{n}^{i}}{2\sqrt{1-\sum_{n}\;[{\left(a_{n}^{i}\right)}^{2}+{\left(b_{n}^{i}\right)}^{2}]/2}}+\text{sin}n\left(\theta -\theta^{i}\right)]+{R\prime }^{i}n\text{cos}n\left(\theta -\theta^{i}\right)}{\sqrt{{\left(R^{i}\right)}^{2}+{\left({R\prime }^{i}\right)}^{2}}}d\theta \\ -\mu_{\mathrm{ab}}\sum_{j\neq i}\;\int_{-\pi }^{\pi }\;d{\theta \phi }^{j}R^{i}\left(\theta \right)R_{0}[-\frac{b_{n}^{i}}{2\sqrt{1-\sum_{n}\;[{\left(a_{n}^{i}\right)}^{2}+{\left(b_{n}^{i}\right)}^{2}]/2}}+\text{sin}n(\theta -\theta^{i})] \end{array}$$

where **e**_θ_ and **e**_⊥_ indicate **e**_θ_ = (cosθ, sinθ) and **e**_⊥_ = (−sinθ, cosθ), while **v***^i^* represents the self-propulsion term **v***^i^* = (*v*_0_ cosθ*^i^*, *v*_0_ sinθ*^i^*), ξ*^i^* denotes a white Gaussian noise with mean zero and variance one, and *R*′*^i^* represents *dR^i^*/*d*θ. Here, ϕ*^j^*(θ) is a function that takes ϕ*^j^*(θ) = 1 when position $u\left(\theta \right)=r_{c}^{i}+R^{i}\left(\theta \right)e_{\theta }$ on the contour in the θ direction of the *i*th cell is occupied by the *j*th cell and takes ϕ*^j^*(θ) = 0 otherwise. This is judged by $R^{j}[\text{arg}\left(u-r_{c}^{j}\right)]>|u-r_{c}^{j}|$, where $u-r_{c}^{j}$ corresponds to a vector from *j*th cell center to the point on the *i*th cell contour and $\text{arg}\left(u-r_{c}^{j}\right)$ represents the angle of the vector against *x* axis. For the numerical implementation, when $\sum_{n=2}^{M}\;\sqrt{{\left(a_{n}^{i}\right)}^{2}+{\left(b_{n}^{i}\right)}^{2}}$ exceeds $\sqrt{2/3}$, $\left\{a_{n}^{i}\right\}$ and $\left\{b_{n}^{i}\right\}$ are normalized to ${\tilde{a}}_{n}^{i}=a_{n}^{i}\sqrt{2/3}/\sum_{n=2}^{M}\sqrt{{\left(a_{n}^{i}\right)}^{2}+{\left(b_{n}^{i}\right)}^{2}}$.

In our simulation, we set μ*_r_* = μ_θ_ = 1 and μ_ab_ = 0.1, and the integral with respect to *d*θ is calculated by discretizing 0 − 2π into 40 points. The other parameters are *D_r_* = 0.01 and *dt* = 0.005. The simulation box size was set as $L_{x}:L_{y}=2:\sqrt{3}$ with the periodic boundary condition. In the numerical calculation, whether ϕ*^j^* = 0 or 1 in the integral can be judged without calculating ϕ*^j^* itself from

$$R_{0}\sqrt{1-\sum_{n=2}^{M}\;\frac{{\left(a_{n}^{j}\right)}^{2}+{\left(b_{n}^{j}\right)}^{2}}{2}}+R_{0}\sum_{n=2}^{M}\;\sqrt{{\left(a_{n}^{j}\right)}^{2}+{\left(b_{n}^{j}\right)}^{2}}<|u-r_{c}^{j}|\;$$ (6)

which can be computed with a lower computational cost when the first and the second terms in the left-hand side are stored in the memory. When the above inequality is not satisfied, then whether ϕ*^j^* = 0 or 1 is determined from $R^{j}[\text{arg}\left(u-r_{c}^{j}\right)]<|u-r_{c}^{j}|$. The detailed derivation of the model is given in Supplementary Text.

### Soft area constraint

Here, a version of the model with a soft area constraint is considered instead of the hard area constraint as was originally assumed. In this case, $a_{0}^{i}$ is also an independent variable. The time evolutions for the variables $r_{c}^{i}$ and θ*^i^* are the same as the original model, whereas those for $a_{n}^{i}$ and $b_{n}^{i}$ are modified as follows

$$\begin{array}{c} {\dot{a}}_{0}^{i}=-\mu_{\mathrm{ab}}\eta \int_{-\pi }^{\pi }\;R_{0}\frac{R^{i}}{\sqrt{{\left(R^{i}\right)}^{2}+{\left({R\prime }^{i}\right)}^{2}}}d\theta \\ -\mu_{\mathrm{ab}}M_{A}[{\left(a_{0}^{i}\right)}^{2}+\sum_{n=2}\;\frac{{\left(a_{n}^{i}\right)}^{2}+{\left(b_{n}^{i}\right)}^{2}}{2}-1]\;2a_{0}^{i}-\mu_{\mathrm{ab}}\sum_{j\neq i}\;\int_{0}^{2\pi }d{\theta \phi }^{j}R^{i}R_{0}\\ {\dot{a}}_{n}^{i}=-\mu_{\mathrm{ab}}\eta \int_{-\pi }^{\pi }\;R_{0}\frac{R^{i}\text{cos}n\left(\theta -\theta^{i}\right)-{R\prime }^{i}n\text{sin}n\left(\theta -\theta^{i}\right)}{\sqrt{{\left(R^{i}\right)}^{2}+{\left({R\prime }^{i}\right)}^{2}}}d\theta \\ -\mu_{\mathrm{ab}}M_{A}[{\left(a_{0}^{i}\right)}^{2}+\sum_{n=2}\;\frac{{\left(a_{n}^{i}\right)}^{2}+{\left(b_{n}^{i}\right)}^{2}}{2}-1]\;a_{n}^{i}-\mu_{\mathrm{ab}}\sum_{j\neq i}\;\int_{0}^{2\pi }\;d{\theta \phi }^{j}R^{i}R_{0}\text{cos}n\left(\theta -\theta^{i}\right)\;\left(\text{for}\;n\geq 2\right)\\ {\dot{b}}_{n}^{i}=-\mu_{\mathrm{ab}}\eta \int_{-\pi }^{\pi }\;R_{0}\frac{R^{i}\text{sin}n\left(\theta -\theta^{i}\right)+{R\prime }^{i}n\text{cos}n\left(\theta -\theta^{i}\right)}{\sqrt{{\left(R^{i}\right)}^{2}+{\left({R\prime }^{i}\right)}^{2}}}d\theta \\ -\mu_{\mathrm{ab}}M_{A}[{\left(a_{0}^{i}\right)}^{2}+\sum_{n=2}\;\frac{{\left(a_{n}^{i}\right)}^{2}+{\left(b_{n}^{i}\right)}^{2}}{2}-1]\;b_{n}^{i}-\mu_{\mathrm{ab}}\sum_{j\neq i}\;\int_{0}^{2\pi }\;d{\theta \phi }^{j}R^{i}R_{0}\text{sin}n\left(\theta -\theta^{i}\right)\;\left(\text{for}\;n\geq 2\right) \end{array}$$

where *M_A_* is a parameter for the area constraint. The detailed derivation of the model is given in Supplementary Text.
