# Supplementary material for: Cell deformability drives fluid-to-fluid phase transition in active cell monolayers
Source: Sci Adv. 2024 May 8;10(19):eadi8433. doi: 10.1126/sciadv.adi8433 (PMC12697573; doi:10.1126/sciadv.adi8433)
Supplement: Supplementary file 1 — Supplementary Text Figs. S1 to S15 Legends for movies S1 to S4 [file sciadv.adi8433_sm.pdf]

Science Advances
AAAS

Supplementary Materials for

Cell deformability drives fluid-to-fluid phase transition in active cell monolayers

Nen Saito and Shuji Ishihara

Corresponding author: Nen Saito, nensaito@hiroshima-u.ac.jp

Sci. Adv. **10**, eadi8433 (2024)
DOI: 10.1126/sciadv.adi8433

The PDF file includes:

Supplementary Text
Figs. S1 to S15
Legends for movies S1 to S4

Other Supplementary Material for this manuscript includes the following:

Movies S1 to S4

I. SUPPLEMENTARY TEXT

A. Model derivation

1. Fourier series description of cell contour

We consider deformable cells interacting in two-dimensional space. We assume that a cell contour can be represented by the polar coordinates using the univariate function $R(\theta)$ (Fig. 1a). The contour $R(\theta)$ of the i-th cell centered at the origin is then expressed by a Fourier expansion up to M-th order as follows:

$$R^i(\theta) = R_0 \left[\sqrt{1 - \sum_{n=2}^{M} \frac{(a_n^i)^2 + (b_n^i)^2}{2}} + \sum_{n=2}^{M} \left(a_n^i \cos n(\theta - \theta^i) + b_n^i \sin n(\theta - \theta^i) \right) \right] \tag{S1}$$

The absence of the first-order coefficients a_1 and b_1 guarantees that the centroid of the cell is fixed at the origin of the expansion. The first term in the above equation also ensures the constant cell area πR_0^2:

$$area = \int_{-\pi}^{\pi} \pi \{R^i(\theta)\}^2 \frac{d\theta}{2\pi} = \pi R_0^2. \tag{S2}$$

To avoid self-crossing of the cell contour, we imposed the constraint $\sum_{n=2}^{M} \sqrt{(a_n^i)^2 + (b_n^i)^2} < \sqrt{2/3}$ that guarantees $R(\theta) > 0$, i.e., the centroid is always located within the cell. This condition also ensures the inside of the square root term in Eq.(S1) is positive. These statements can be confirmed by the following inequalities,

$$\begin{aligned} R^i(\theta)/R_0 &\geq \sqrt{1 - \sum_{n=2}^{M} \frac{(a_n^i)^2 + (b_n^i)^2}{2}} - \sum_{n=2}^{M} \sqrt{(a_n^i)^2 + (b_n^i)^2} \\ &\geq \sqrt{1 - \frac{1}{2} \left(\sum_{n=2}^{M} \sqrt{(a_n^i)^2 + (b_n^i)^2} \right)^2} - \sum_{n=2}^{M} \sqrt{(a_n^i)^2 + (b_n^i)^2} \\ &= \frac{1 - \frac{3}{2} \left(\sum_{n=2}^{\infty} \sqrt{(a_n^i)^2 + (b_n^i)^2} \right)^2}{\sqrt{1 - \frac{1}{2} \left(\sum_{n=2}^{\infty} \sqrt{(a_n^i)^2 + (b_n^i)^2} \right)^2} + \sum_{n=2} \sqrt{(a_n^i)^2 + (b_n^i)^2}} \\ &> 0 \end{aligned} \tag{S3}$$

and

$$1 - \sum_{n=2}^{M} \frac{(a_n^i)^2 + (b_n^i)^2}{2} \geq 1 - \frac{1}{2} \left(\sum_{n=2}^{M} \sqrt{(a_n^i)^2 + (b_n^i)^2} \right)^2 > 0. \tag{S4}$$

For the numerical implementation, when $\sum_{n=2}^{M} \sqrt{(a_n^i)^2 + (b_n^i)^2}$ exceeds $\sqrt{2/3}$, $\{a_n^i\}$ and $\{b_n^i\}$ are normalized to $\tilde{a}_n^i = a_n^i \sqrt{2/3} / \sum_{n=2}^{M} \sqrt{(a_n^i)^2 + (b_n^i)^2}$.

2. Hamiltonian

The total Hamiltonian of the model is given by

$$\mathcal{H} = \mathcal{H}_l + \mathcal{H}_{int} = \sum_i \mathcal{H}_l^i + \sum_{i<j} \mathcal{H}_{int}^{ij}, \tag{S5}$$

where $\mathcal{H}_l$ represents the energy for penalizing interface length associated with the membrane tension, $\mathcal{H}_{int}$ denotes the interaction term through the excluded volume effect, and $\sum_{i<j}$ is the summation for all i-j pairs. The interfacial energy for each cell is given by

$$\mathcal{H}_l^i = \eta l^i, \tag{S6}$$

where η is the surface tension and l^i represents the length of the contour of i-th cell, which is given by

$$l^i = \int_{-\pi}^{\pi} \sqrt{(R^i)^2 + (R^i)'^2} d\theta. \tag{S7}$$

Note that $(R^i)' = dR^i/d\theta$ in the above equation. The contributions of $\mathcal{H}_l^i$ to the time evolution of a_n^i and b_n^i are denoted by F_{l,a_n}^i and F_{l,b_n}^i, respectively, and are calculated by

$$F_{l,a_n}^i = -\mu_{ab}\frac{d\mathcal{H}_l^i}{da_n^i} = -\mu_{ab}\eta\frac{\partial l^i}{\partial a_n^i} \tag{S8}$$

$$F_{l,b_n}^i = -\mu_{ab}\frac{d\mathcal{H}_l^i}{db_n^i} = -\mu_{ab}\eta\frac{\partial l^i}{\partial b_n^i}, \tag{S9}$$

where μ_{ab} is the mobility parameter for a_n and b_n. These derivatives $\partial l^i/\partial a_n^i$ and $\partial l^i/\partial b_n^i$ are further calculated as follows:

$$\begin{aligned}
\frac{\partial l^i}{\partial a_n^i} &= \int_{-\pi}^{\pi} \frac{R^i\frac{\partial R^i}{\partial a_n^i} + R'^i\frac{\partial R'^i}{\partial a_n^i}}{\sqrt{(R^i)^2+(R'^i)^2}} d\theta \\
&= \int_{-\pi}^{\pi} R_0 \frac{R^i\left(-\frac{a_n^i}{2\sqrt{1-\sum_n((a_n^i)^2+(b_n^i)^2)/2}} + \cos n(\theta-\theta^i)\right) - R'^i n\sin n(\theta-\theta^i)}{\sqrt{(R^i)^2+(R'^i)^2}} d\theta
\end{aligned} \tag{S10}$$

$$\begin{aligned}
\frac{\partial l^i}{\partial b_n^i} &= \int_{-\pi}^{\pi} \frac{R^i\frac{\partial R^i}{\partial b_n^i} + R'^i\frac{\partial R'^i}{\partial b_n^i}}{\sqrt{(R^i)^2+(R'^i)^2}} d\theta \\
&= \int_{-\pi}^{\pi} R_0 \frac{R^i\left(-\frac{b_n^i}{2\sqrt{1-\sum_n((a_n^i)^2+(b_n^i)^2)/2}} + \sin n(\theta-\theta^i)\right) + R'^i n\cos n(\theta-\theta^i)}{\sqrt{(R^i)^2+(R'^i)^2}} d\theta.
\end{aligned} \tag{S11}$$

Other expressions of $\partial l^i/\partial a_n^i$ and $\partial l^i/\partial b_n^i$ are given as $\partial l^i/\partial a_n^i = \int_{-\pi}^{\pi}\kappa(\theta)R^i\frac{\partial R^i}{\partial a_n^i}d\theta$ and $\partial l^i/\partial b_n^i = \int_{-\pi}^{\pi}\kappa(\theta)R^i\frac{\partial R^i}{\partial b_n^i}d\theta$ respectively, where $\kappa = \frac{-R''R+2(R')^2+R^2}{((R')^2+R^2)^{3/2}}$ represents the local curvature.

The interaction energy $\mathcal{H}_{int}$ through the excluded volume effect is formulated as

$$\mathcal{H}_{int} = \sum_{i>j}\int d\boldsymbol{r}\phi^i\phi^j \tag{S12}$$

by using the field representation of the cell in Eq. (2) in the main text. Contribution of $\mathcal{H}_{int}$ to the dynamics in $\boldsymbol{r}_c^i$ is summarized as the force terms $\boldsymbol{F}_{int,r}^i$, which are given as

$$\begin{aligned}
\boldsymbol{F}_{int,r}^i = -\mu_r\frac{d\mathcal{H}_{int}}{d\boldsymbol{r}_c^i} &= \mu_r\int d\boldsymbol{r}\frac{\delta\mathcal{H}_{int}}{\delta\phi^i}\nabla\phi^i \\
&= \mu_r\sum_{j\neq i}\int d\boldsymbol{r}\phi^j\nabla\phi^i(\boldsymbol{r}) \\
&= \mu_r\sum_{j\neq i}\int d\boldsymbol{r}\phi^j|\nabla\phi^i(\boldsymbol{r})|\boldsymbol{n}^i(\boldsymbol{r}),
\end{aligned} \tag{S13}$$

where μ_r indicate the mobility parameter for $\boldsymbol{r}_c$. In the above equation, $\boldsymbol{n}^i = \nabla\phi^i/|\nabla\phi^i|$ represents the normal vector

to the cell contour and is given by

$$
\begin{aligned}
\boldsymbol{n}^i(\boldsymbol{r}) &= \frac{\nabla\left(R^i(\hat{\theta}) - \Delta^i(\boldsymbol{r},\boldsymbol{r}_c^i)\right)}{|\nabla\left(R^i(\hat{\theta}) - \Delta^i(\boldsymbol{r},\boldsymbol{r}_c^i)\right)|} \\
&= \frac{\left(R'^i(\hat{\theta})\nabla\hat{\theta} - \nabla|\boldsymbol{r}-\boldsymbol{r}_c^i|\right)}{|\left(R'^i(\hat{\theta})\nabla\hat{\theta} - \nabla|\boldsymbol{r}-\boldsymbol{r}_c^i|\right)|} \\
&= \frac{\left(R'^i\frac{\boldsymbol{r}_\perp}{|\boldsymbol{r}-\boldsymbol{r}_c^i|^2} - \frac{\boldsymbol{r}-\boldsymbol{r}_c^i}{|\boldsymbol{r}-\boldsymbol{r}_c^i|}\right)}{\sqrt{1+\left(\frac{R'^i}{R^i}\right)^2}},
\end{aligned}
\tag{S14}
$$

where $\hat{\theta}$ is the angle between $\boldsymbol{r}-\boldsymbol{r}_c^i$ and x axis, and $\boldsymbol{r}_\perp$ is the vector perpendicular to $\boldsymbol{r}-\boldsymbol{r}_c^i$ as $\boldsymbol{r}_\perp = (-y+y_c, x-x_c)$. By considering the sharp interface limit $\epsilon \to 0$, $|\nabla\phi^i(\boldsymbol{r})|$ can be considered as the surface delta function, which leads to $\int f(\boldsymbol{r})|\nabla\phi^i(\boldsymbol{r})|d\boldsymbol{r} = \int f(\boldsymbol{r}(s))ds$. In the integral $\int f(\boldsymbol{r})|\nabla\phi^i(\boldsymbol{r})|d\boldsymbol{r}$, only $\boldsymbol{r}$ that satisfies $R^i(\hat{\theta}) - \Delta^i(\boldsymbol{r},\boldsymbol{r}_c^i) = 0$, i.e., $\boldsymbol{r} = \boldsymbol{r}_c + R^i(\hat{\theta})(\cos\hat{\theta},\sin\hat{\theta})^T$ contributes to the integral, and thus the two-dimensional integral $\int d\boldsymbol{r}$ is replaced by the line integral along the cell contour $\oint ds = \int_0^{2\pi}\sqrt{R^i(\theta)^2 + R'^i(\theta)^2 d\theta}$, where $\hat{\theta}$ is no longer a function of $\boldsymbol{r}$ but the integral variable θ.

Using $\boldsymbol{e}_\theta = (\cos\theta, \sin\theta)$ and $\boldsymbol{e}_\perp = (-\sin\theta, \cos\theta)$, $\boldsymbol{F}_{int,r}^i$ is calculated as:

$$
\begin{aligned}
\boldsymbol{F}_{int,r}^i &= \mu_r \sum_{j\neq i}\int ds\phi^j\boldsymbol{n}^i(\boldsymbol{r}) \\
&= \mu_r \sum_{j\neq i}\int_0^{2\pi} ds\phi^j\frac{\frac{R'^i}{R^i}\boldsymbol{e}_\perp - \boldsymbol{e}_\theta}{\sqrt{1+\left(\frac{R'^i}{R^i}\right)^2}} \\
&= \mu_r \sum_{j\neq i}\int_0^{2\pi} d\theta\phi^j\left(R'^i(\theta)\boldsymbol{e}_\perp - R^i(\theta)\boldsymbol{e}_\theta\right)
\end{aligned}
\tag{S15}
$$

Here, $\phi^j(\theta)$ is a function that takes $\phi^j(\theta) = 1$ when position $\boldsymbol{u}(\theta) = \boldsymbol{r}_c^i + R(\theta)\boldsymbol{e}_\theta$ on the contour in the θ direction of the i-th cell, is occupied by the j-th cell, and takes $\phi^j(\theta) = 0$ otherwise. This is judged by $R^j(\theta'(\boldsymbol{u}-\boldsymbol{r}_c^j)) > |\boldsymbol{u}-\boldsymbol{r}_c^j|$, where $\boldsymbol{u}-\boldsymbol{r}_c^j$ corresponds to a vector from j-th cell centroid to the point on the i-th cell contour and $\theta'(\boldsymbol{u}-\boldsymbol{r}_c^j)$ represents the angle of the vector against x axis. By introducing $\boldsymbol{f}_r^{i,j}(\theta)$, the force caused by collision between ith and jth cell on the θ direction of ith cell, $\boldsymbol{F}_{int,r}^i$ is also written as

$$
\boldsymbol{F}_{int,r}^i = \mu_r \sum_{j\neq i}\int_0^{2\pi} d\theta\boldsymbol{f}_r^{i,j}(\theta), \tag{S16}
$$

where $\boldsymbol{f}_r^{i,j}(\theta) = \phi^j\left(R'^i(\theta)\boldsymbol{e}_\perp - R^i(\theta)\boldsymbol{e}_\theta\right)$.

Similarly, the contribution of the interaction Hamiltonian $\mathcal{H}_{int}$ to the dynamics of θ^i is denoted as $F_{int,\theta}^i$ and

calculated by:

$$\begin{aligned}
F^i_{int,\theta} &= -\mu_\theta \frac{d\mathcal{H}}{d\theta^i} = -\mu_\theta \int d\boldsymbol{r} \frac{\delta\mathcal{H}}{\delta\phi^i}\frac{\partial\phi^i}{\partial\theta^i} \\
&= -\mu_\theta \sum_{i\neq j}\int d\boldsymbol{r}\phi^j|\nabla\phi^i(\boldsymbol{r})|\frac{\partial_{\theta^i}\phi^i(\boldsymbol{r})}{|\nabla\phi^i(\boldsymbol{r})|} \\
&= -\mu_\theta \sum_{i\neq j}\int d\boldsymbol{r}\phi^j|\nabla\phi^i(\boldsymbol{r})|\frac{\partial_{\theta^i}\left(R^i(\hat\theta)-\Delta^i(\boldsymbol{r},\boldsymbol{r}^i_c)\right)}{|\nabla\left(R^i(\hat\theta)-\Delta^i(\boldsymbol{r},\boldsymbol{r}^i_c)\right)|} \\
&= \mu_\theta \sum_{i\neq j}\int d\boldsymbol{r}\phi^j|\nabla\phi^i(\boldsymbol{r})|\frac{\frac{\partial R^i(\hat\theta)}{\partial\hat\theta}}{|\left(\frac{\partial R(\hat\theta)}{\partial\hat\theta}\nabla\hat\theta-\nabla|\boldsymbol{r}-\boldsymbol{r}^i_c|\right)|} \\
&= \mu_\theta \sum_{i\neq j}\int d\boldsymbol{r}\phi^j|\nabla\phi^i(\boldsymbol{r})|\frac{R'^i(\hat\theta)}{\sqrt{1+\left(\frac{R'^i}{R^i}\right)^2}} \\
&= \mu_\theta \sum_{i\neq j}\int ds\phi^j\frac{R'^i}{\sqrt{1+\left(\frac{R'^i}{R^i}\right)^2}} \\
&= \mu_\theta \sum_{i\neq j}\int d\theta\phi^j R'^i(\theta)R^i(\theta),
\end{aligned} \tag{S17}$$

where μ_θ represents the mobility parameter for θ^i. Note that $F^i_{int,\theta}$ can be represented by using $\boldsymbol{f}^{i,j}_r(\theta)$ as:

$$F^i_{int,\theta} = \mu_\theta \sum_{j\neq i}\int_0^{2\pi}(\boldsymbol{r}(\theta)-\boldsymbol{r}^i_c)\times\boldsymbol{f}^{i,j}_r(\theta)\ \ d\theta \tag{S18}$$

where $\boldsymbol{r}(\theta)$ is a point on the i-th cell contour in θ direction, $\boldsymbol{r}^i_c$ is the cell centroid, and $\times$ represents the outer product of the vectors. This formula merely denotes that the term is the torque caused by the collision between i-th and j-th cells. The mobility parameter μ_θ represents the inverse of the rotational friction. When i-th and j-th cells are elongated along the self-propulsion direction, this torque term can operate like an alignment interaction (as is similar to the self-propelled rod), whereas this is not the case for laterally elongated cells.

Similarly, the contributions of $\mathcal{H}_{int}$ to the time evolution of a^i_n and b^i_n are denoted as the terms F^i_{int,a_n} and F^i_{int,b_n} and are also given with the mobility parameter μ_{ab} as:

$$\begin{aligned}
F^i_{int,a_n} &= -\mu_{ab}\frac{d\mathcal{H}_{int}}{da^i_n} \\
&= -\mu_{ab}\sum_{j\neq i}\int d\boldsymbol{r}\phi^j|\nabla\phi^i(\boldsymbol{r})|\frac{\partial_{a^i_n}\phi^i(\boldsymbol{r})}{|\nabla\phi^i(\boldsymbol{r})|} \\
&= -\mu_{ab}\sum_{j\neq i}\int d\boldsymbol{r}\phi^j|\nabla\phi^i(\boldsymbol{r})|\frac{\frac{\partial R^i(\hat\theta)}{\partial a^i_n}}{\sqrt{1+\left(\frac{R'^i}{R^i}\right)^2}} \\
&= -\mu_{ab}\sum_{j\neq i}\int d\boldsymbol{r}\phi^j|\nabla\phi^i(\boldsymbol{r})|R_0\frac{\left(-\frac{a^i_n}{2\sqrt{1-\sum_n((a^i_n)^2+(b^i_n)^2)/2}}+\cos(n\hat\theta-n\theta^i)\right)}{\sqrt{1+\left(\frac{R'^i}{R^i}\right)^2}} \\
&= -\mu_{ab}\sum_{j\neq i}\int d\theta\phi^j R^i(\theta)R_0\left(-\frac{a^i_n}{2\sqrt{1-\sum_n((a^i_n)^2+(b^i_n)^2)/2}}+\cos(n\theta-n\theta^i)\right)
\end{aligned} \tag{S19}$$

and,

$$F^i_{int,b_n} = -\mu_{ab}\sum_{j\neq i}\int d\theta\phi^j R^i(\theta)R_0\left(-\frac{b^i_n}{2\sqrt{1-\sum_n((a^i_n)^2+(b^i_n)^2)/2}}+\sin(n\theta-n\theta^i)\right) \tag{S20}$$

B. Time evolution equation

By summing up all the force terms, the time evolution equations for $\boldsymbol{r}^i_c$, θ^i, a^i_n and b^i_n are obtained as follows:

$$\dot{\boldsymbol{r}}^i_c = \boldsymbol{v}^i + \boldsymbol{F}^i_{int,r} = \boldsymbol{v}^i + \mu_r\sum_{j\neq i}\int_{-\pi}^{\pi} d\theta\phi^j\left(R'^i(\theta)\boldsymbol{e}_\perp - R^i(\theta)\boldsymbol{e}_\theta\right) \tag{S21}$$

$$\dot{\theta}^i = F^i_{int,\theta} + \sqrt{2D_r}\xi^i = \mu_\theta\sum_{j\neq i}\int_{-\pi}^{\pi} d\theta\phi^j R'^i(\theta)R^i(\theta) + \sqrt{2D_r}\xi^i \tag{S22}$$

$$\begin{aligned}\dot{a}^i_n &= F^i_l + F^i_{int,a_n}\\ &= -\mu_{ab}\eta\int_{-\pi}^{\pi} R_0\frac{R^i\left(-\frac{a^i_n}{2\sqrt{1-\sum_n((a^i_n)^2+(b^i_n)^2)/2}}+\cos n(\theta-\theta^i)\right) - R'^i n\sin n(\theta-\theta^i)}{\sqrt{(R^i)^2+(R'^i)^2}}d\theta\\ &- \mu_{ab}\sum_{j\neq i}\int_{-\pi}^{\pi} d\theta\phi^j R^i(\theta)R_0\left(-\frac{a^i_n}{2\sqrt{1-\sum_n((a^i_n)^2+(b^i_n)^2)/2}}+\cos(n\theta-n\theta^i)\right)\end{aligned} \tag{S23}$$

$$\begin{aligned}\dot{b}^i_n &= F^i_l + F^i_{int,b_n}\\ &= -\mu_{ab}\eta\int_{-\pi}^{\pi} R_0\frac{R^i\left(-\frac{b^i_n}{2\sqrt{1-\sum_n((a^i_n)^2+(b^i_n)^2)/2}}+\sin n(\theta-\theta^i)\right) + R'^i n\cos n(\theta-\theta^i)}{\sqrt{(R^i)^2+(R'^i)^2}}d\theta\\ &- \mu_{ab}\sum_{j\neq i}\int_{-\pi}^{\pi} d\theta\phi^j R^i(\theta)R_0\left(-\frac{b^i_n}{2\sqrt{1-\sum_n((a^i_n)^2+(b^i_n)^2)/2}}+\sin(n\theta-n\theta^i)\right),\end{aligned} \tag{S24}$$

where $\boldsymbol{v}^i$ represents the self-propulsion term $\boldsymbol{v}^i = (v_0\cos\theta^i, v_0\sin\theta^i)$ and ξ^i denotes a normalized white Gaussian noise. In our simulation, we set $\mu_r = \mu_\theta = 1$ and $\mu_{ab} = 0.1$, and the integral with respect to $d\theta$ is calculated by discretizing 0 - 2π into 40 points if not mentioned otherwise. The other parameters are $D_r = 0.01$ and $dt = 0.005$. In the numerical calculation in the integral $\int d\theta\phi^j[\cdots]$, $\phi^j = 0$ can be judged without calculating ϕ^j itself from

$$\sqrt{1-\sum_{n=2}^{M}\frac{(a^j_n)^2+(b^j_n)^2}{2}} + \sum_{n=2}^{M}\sqrt{(a^j_n)^2+(b^j_n)^2} < |\boldsymbol{u}-\boldsymbol{r}^j_c|, \tag{S25}$$

which can be computed with a lower computational cost when the first and the second terms are stored in the memory. When the above inequality is not satisfied, then whether $\phi^j = 0$ or 1 is determined from $R^j(\theta'(\boldsymbol{u}-\boldsymbol{r}^j_c)) < |\boldsymbol{u}-\boldsymbol{r}^j_c|$.

C. Soft Area Constraint

A version of the model with a soft area constraint is considered instead of the hard area constraint.

1. *Hamiltonians*

The same $R(\theta)$ to the original model (Eq. (1) in the main text) is considered as

$$R^i(\theta) = R_0\left[a^i_0 + \sum_{n=1}^{\infty}\left(a^i_n\cos n(\theta-\theta^i) + b^i_n\sin n(\theta-\theta^i)\right)\right], \tag{S26}$$

where a_0^i is now an independent variable rather than a function of a_n^i and b_n^i. Other conditions were set to behave in the same manner as the original model. The constraint that the center of the polar coordinate coincides with the cell centroid requires $a_1^i = b_1^i = 0$, whereas we also impose $\sum_{n=2}^{M} \sqrt{(a_n^i)^2 + (b_n^i)^2} < \sqrt{2/3}$ and $a_0^i > \sqrt{2/3}$ for avoiding self-crossing. The total Hamiltonian is given as follows:

$$\mathcal{H} = \mathcal{H}_l + \mathcal{H}_A + \mathcal{H}_{int} \tag{S27}$$

where $\mathcal{H}_l$ is an energy cost for having a long cell perimeter, $\mathcal{H}_A$ is that for the soft area constraint, and $\mathcal{H}_{int}$ is that for the excluded volume interactions.

The energy cost for having a long perimeter is given by the same expression as the original model:

$$\mathcal{H}_l = \eta \sum_i l^i = \eta \sum_i \int_{-\pi}^{\pi} \sqrt{(R^i(\theta))^2 + (R^{i\prime}(\theta))^2} d\theta, \tag{S28}$$

where η is the tension parameter. Contribution of the $\mathcal{H}_l$ to the dynamics in variables $\boldsymbol{r}_c^i, \theta^i, a_n^i, b_n^i$ are denoted by $\boldsymbol{F}_{l,r}^i, F_{l,\theta}^i, F_{l,a_n}^i$ and F_{l,b_n}^i as

$$\boldsymbol{F}_{l,r}^i = 0 \tag{S29}$$

$$F_{l,\theta}^i = -\mu_\theta \int_{-\pi}^{\pi} \frac{\delta \mathcal{H}_l}{\delta \theta^i} d\theta = \mu_\theta \eta \int_{-\pi}^{\pi} -\frac{\partial}{\partial \theta} \sqrt{(R^i)^2 + (R^{i\prime})^2} d\theta = 0 \tag{S30}$$

$$\begin{aligned} F_{l,a_n}^i &= -\mu_{ab} \int_{-\pi}^{\pi} \frac{\delta \mathcal{H}_l}{\delta a_n^i} d\theta = -\mu_{ab}\eta \int_{-\pi}^{\pi} \frac{R^i \frac{\partial R^i}{\partial a_n^i} + R^{i\prime} \frac{\partial R^{i\prime}}{\partial a_n^i}}{\sqrt{(R^i)^2 + (R^{i\prime})^2}} d\theta = -\mu_{ab}\eta \int_{-\pi}^{\pi} \kappa^i R^i \frac{\partial R^i}{\partial a_n^i} d\theta \\ &= -\mu_{ab}\eta \int_{-\pi}^{\pi} \kappa^i R^i \cos n(\theta - \theta^i) d\theta \end{aligned} \tag{S31}$$

$$\begin{aligned} F_{l,b_n}^i &= -\mu_{ab} \int_{-\pi}^{\pi} \frac{\delta \mathcal{H}_l}{\delta b_n^i} d\theta = -\mu_{ab}\eta \int_{-\pi}^{\pi} \frac{R^i \frac{\partial R^i}{\partial b_n^i} + R^{i\prime} \frac{\partial R^{i\prime}}{\partial b_n^i}}{\sqrt{(R^i)^2 + (R^{i\prime})^2}} d\theta = -\mu_{ab}\eta \int_{-\pi}^{\pi} \kappa^i R^i \frac{\partial R^i}{\partial b_n^i} d\theta \\ &= -\mu_{ab}\eta \int_{-\pi}^{\pi} \kappa^i R^i \sin n(\theta - \theta^i) d\theta, \end{aligned} \tag{S32}$$

where $\kappa^i = \frac{-R^{i\prime\prime} R^i + 2(R^{i\prime})^2 + (R^i)^2}{((R^{i\prime})^2 + (R^i)^2)^{3/2}}$ represents the local curvature.

The energy for the soft area constraint $\mathcal{H}_A$ denotes an energy penalty to keep the cell area $A = \pi R_0^2 \left[a_0^2 + \sum_{n=2} \frac{a_n^2 + b_n^2}{2} \right]$ constant, and is given as follows:

$$\mathcal{H}_A = \sum_i \frac{M_A}{2\pi R_0^2} (A^i - A_0)^2 = \sum_i \frac{M_A}{2} \left((a_0^i)^2 + \sum_{n=2} \frac{(a_n^i)^2 + (b_n^i)^2}{2} - 1 \right)^2, \tag{S33}$$

where A_0 is the target cell area $A_0 = \pi R_0^2$, and M_A represents a penalty parameter for the soft area constraint. Contribution of $\mathcal{H}_A$ to the dynamics in $\boldsymbol{r}_c^i, \theta^i, a_n^i, b_n^i$ are summarized as the terms $\boldsymbol{F}_{A,r}^i$, $F_{A,\theta}^i, F_{A,a_n}^i, F_{A,b_n}^i$, which are given as

$$\boldsymbol{F}_{A,r}^i = 0 \tag{S34}$$

$$F_{A,\theta}^i = 0 \tag{S35}$$

$$F_{A,a_0}^i = -\mu_{ab} \frac{\partial \mathcal{H}_A}{\partial a_0^i} d\theta = -\mu_{ab} M_A \left((a_0^i)^2 + \sum_{n=2} \frac{(a_n^i)^2 + (b_n^i)^2}{2} - 1 \right) 2a_0^i \tag{S36}$$

$$F_{A,a_n}^i = -\mu_{ab} \frac{\partial \mathcal{H}_A}{\partial a_n^i} d\theta = -\mu_{ab} M_A \left((a_0^i)^2 + \sum_{n=2} \frac{(a_n^i)^2 + (b_n^i)^2}{2} - 1 \right) a_n^i \quad \text{for} \quad n \geq 2 \tag{S37}$$

$$F_{A,b_n}^i = -\mu_{ab} \frac{\partial \mathcal{H}_A}{\partial b_n^i} d\theta = -\mu_{ab} M_A \left((a_0^i)^2 + \sum_{n=2} \frac{(a_n^i)^2 + (b_n^i)^2}{2} - 1 \right) b_n^i \quad \text{for} \quad n \geq 2 \tag{S38}$$

(S39)

The Hamiltonian for the excluded volume effect between cells, $\mathcal{H}_{int}$, is given by

$$\mathcal{H}_{int} = \sum_{i>j} \int \phi^i(\boldsymbol{r})\phi^j(\boldsymbol{r})d\boldsymbol{r}, \tag{S40}$$

where

$$\phi^i(\boldsymbol{r}) = \frac{1 + \tanh(\frac{R(\hat{\theta}(\boldsymbol{r},\boldsymbol{r}_c^{(i)})) - \Delta R}{\epsilon/2})}{2}. \tag{S41}$$

The derived terms that contribute to the dynamics in $\boldsymbol{r}_c^i, \theta^i, a_n^i, b_n^i$ are given as

$$\boldsymbol{F}^i_{int,r} = \sum_{j\neq i} \int_0^{2\pi} d\theta \phi^j \left(R^{i\prime}(\theta)\boldsymbol{e}_\perp - R^i(\theta)\boldsymbol{e}_\theta \right) \tag{S42}$$

$$F^i_{int,\theta} = \sum_{j\neq i} \int_0^{2\pi} d\theta \phi^j R^i(\theta) R^{i\prime}(\theta) \tag{S43}$$

$$F^i_{int,a_n} = -\sum_{j\neq i} \int_0^{2\pi} d\theta \phi^j R^i(\theta) \frac{\partial R^i(\theta)}{\partial a_n^i} \tag{S44}$$

$$F^i_{int,b_n} = -\sum_{j\neq i} \int_0^{2\pi} d\theta \phi^j R^i(\theta) \frac{\partial R^i(\theta)}{\partial b_n^i}. \tag{S45}$$

2. Dynamics

From the above, the time evolution of variables $\boldsymbol{r}_c^i, \theta^i, a_n^i, b_n^i$ is obtained as

$$\dot{\boldsymbol{r}}_c^i = \boldsymbol{v}^i + \mu_r \sum_{j\neq i} \int_0^{2\pi} d\theta \phi^j \left(R^{i\prime}(\theta)\boldsymbol{e}_\perp - R^i(\theta)\boldsymbol{e}_\theta \right)$$

$$\dot{\theta}^i = \mu_\theta \sum_{j\neq i} \int_0^{2\pi} d\theta \phi^j R^i(\theta) R^{i\prime}(\theta) + \sqrt{2D_r}\xi^i$$

$$\dot{a}_0^i = \mu_{ab} \left(-\eta \int_{-\pi}^{\pi} \kappa^i R^i R_0 d\theta - M_A \left((a_0^i)^2 + \sum_{n=2} \frac{(a_n^i)^2 + (b_n^i)^2}{2} - 1 \right) 2a_0^i - \sum_{j\neq i} \int_0^{2\pi} d\theta \phi^j R^i R_0 \right)$$

$$\dot{a}_n^i = \mu_{ab} \left(-\eta \int_{-\pi}^{\pi} \kappa^i R^i R_0 \cos n(\theta - \theta^i) d\theta - M_A \left((a_0^i)^2 + \sum_{n=2} \frac{(a_n^i)^2 + (b_n^i)^2}{2} - 1 \right) a_n^i - \sum_{j\neq i} \int_0^{2\pi} d\theta \phi^j R^i R_0 \cos n(\theta - \theta^i) \right)$$

$$\dot{b}_n^i = \mu_{ab} \left(-\eta \int_{-\pi}^{\pi} \kappa^i R^i R_0 \sin n(\theta - \theta^i) d\theta - M_A \left((a_0^i)^2 + \sum_{n=2} \frac{(a_n^i)^2 + (b_n^i)^2}{2} - 1 \right) b_n^i - \sum_{j\neq i} \int_0^{2\pi} d\theta \phi^j R^i R_0 \sin n(\theta - \theta^i) \right)$$

D. Multicellular phase-field model

For comparison between the proposed Fourier contour model and the multi-phase field model, we implemented a two-dimensional multicellular phase-field model combining Nonomura [25] and Loewe et al. [14]. The system is described by the phase-fields variables $\phi_i(t, \mathbf{r})$ $(i = 1, 2, \cdots, N)$ that represents the i-th cellular shape; $\phi_i = 1$ indicates inner region of i-th cell, while $\phi_i = 0$ does the outer region. The free energy is introduced as [25]

$$F[\{\phi_i\}] = \sum_i \int G(\phi_i) d\Omega + \sum_i \frac{K}{2}\left(1 - \frac{A_i}{A_0}\right)^2 + \sum_{i \neq j} \beta \int h(\phi_i) h(\phi_j) d\Omega \tag{S46}$$

Here $d\Omega = dxdy$ and $h(\phi) = \phi^2(3 - 2\phi)$. In the first term $G(\phi) = \frac{\alpha}{4}\phi^2(1-\phi)^2 + \frac{D}{2}|\nabla\phi|^2$ is the basal energy density function for phase-filed. In the second term, $A_i \equiv \int h(\phi_i) dA$ is the cell area of i-th cell, and this term provides the cell area elasticity, for which A_0 is the preferential cell area. The third term indicates the excluded volume effect between cells, which becomes higher when two cells are overlapped. We solved the following equation for active multi phase-filed model:

$$\frac{\partial \phi_i}{\partial t} + v_i \cdot \nabla \phi_i = -\Gamma \frac{\delta F}{\delta \phi_i} = \Gamma\left(D\nabla^2\phi_i + \alpha\phi_i(1-\phi_i)\left(\phi_i - \frac{1}{2} + f_i\right)\right) \tag{S47}$$

$$f_i = K\left(1 - \frac{A_i}{A_0}\right) - \beta \sum_{j \neq i} h(\phi_j) \tag{S48}$$

Here f_i describes forces by cell area elasticity and the excluded volume effect. $v_i = v_0 (\cos\theta_i(t), \sin\theta_i(t))$ is introduced as self-propelled velocity of the i-th cell [14], where the direction $\theta_i(t)$ obeys

$$\frac{d\theta_i}{dt} = \sqrt{2D_r}\xi_i \tag{S49}$$

with normal white Gaussian noise $\xi_i(t)$.

In the simulation, $N = 50, 100, 200$, and 400 cells were prepared on 372×321, 525×453, 741×639, and 1050×906 lattice. Note that, following the method in [25], each phase-filed ϕ_i was simulated on a smaller 123×123 lattice. Space and time are discretized as $\Delta x = 1/3$ (i.e., the system size is 124×107 for $N = 50$, 175×151 for $N = 100$, 247×213 for $N = 200$ and 350×302 for $N = 400$) and $\Delta t = 1.0 \times 10^{-3}$. Time evolution was carried out by the explicit Euler method. For the discretization of Laplacian, the second nearest neighbors were taken into account while the upwind derivative was used for the advection term. Parameters were chosen as $\Gamma = 2.0$, $\alpha = 15.0$, $D = \alpha(\Delta x)^2/2$, $A_0 = \pi(8.2)^2$, $K = 5.0 \times 10^4$, $\beta = 30.0$, $v_0 = 0.35$, and $D_r = 0.04$.

II. SUPPLEMENTAL FIGURES

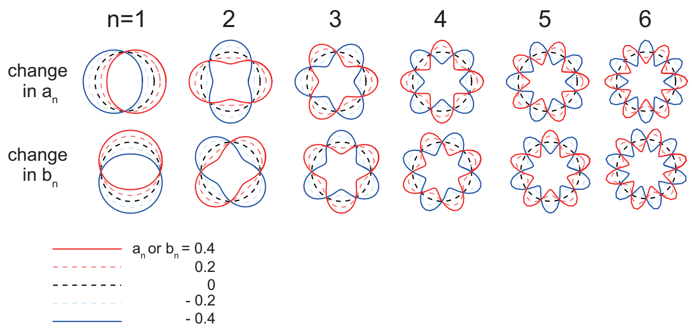

FIG. S1: Visualization of the role of each Fourier mode in the cell shape. The top panels represent the shape changes by the change in a_n from the perfect circular shape $a_0 = 1$ and $a_n = b_n = 0$, whereas the bottom panels indicate the shape changes by the change in b_n.

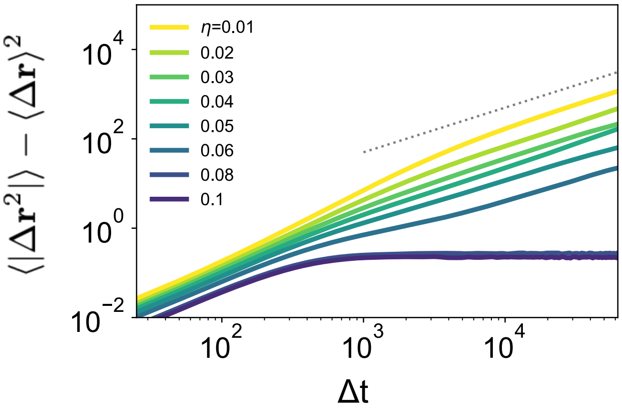

FIG. S2: The mean square displacement measured from the centroid of the cell population for $v = 0.03$. The MSD is calculated by $MSD(t) = \left(\langle(\Delta r)^2\rangle - \langle\Delta r\rangle^2\right)$, where Δr represents the displacement during Δt. This is evaluated from simulation with $N = 1024$ and 8×10^7 time steps with $dt = 0.005$ and $D_r = 0.01$. The dotted black line indicates $y \propto \Delta t$ (i.e., diffusion).

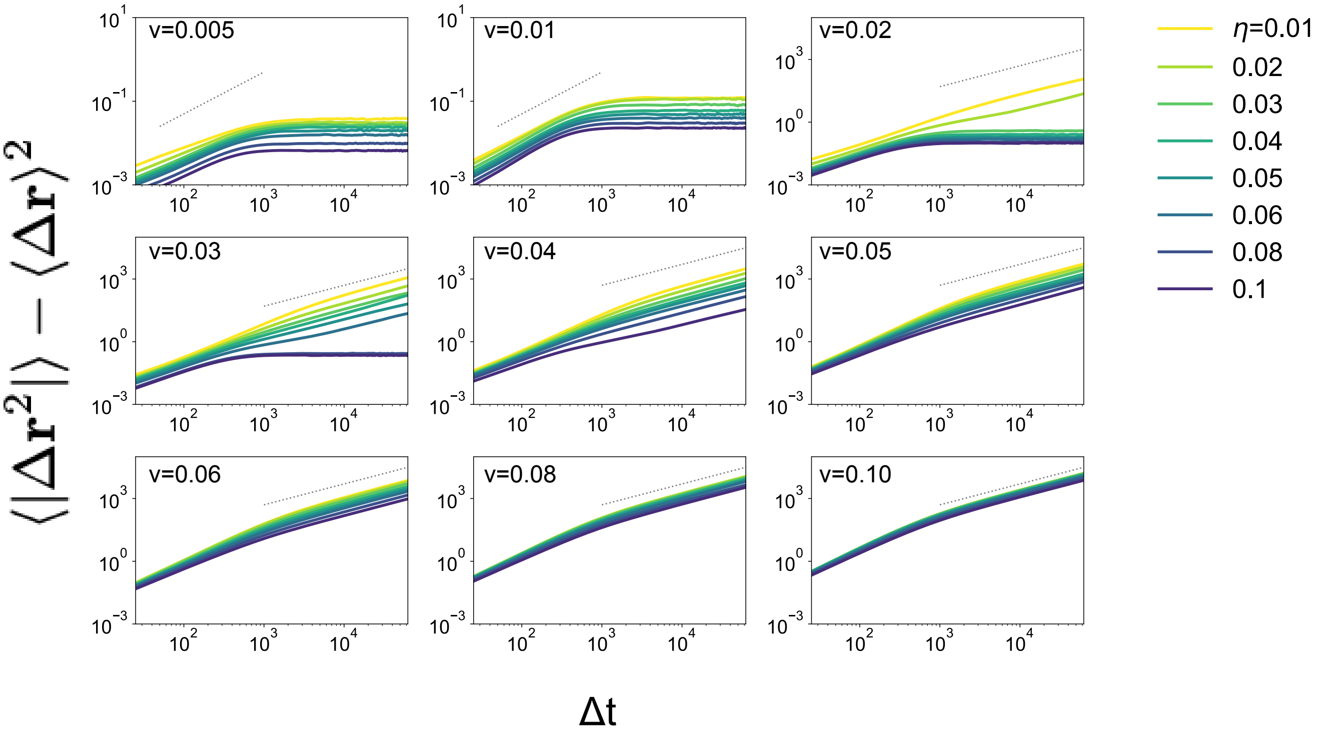

FIG. S3: The mean square displacement measured from the centroid of the cell population for all v and η. The panel for $v = 0.03$ is the same figure as fig. S2. The dotted black line indicates $y \propto \Delta t$ (i.e., diffusion).

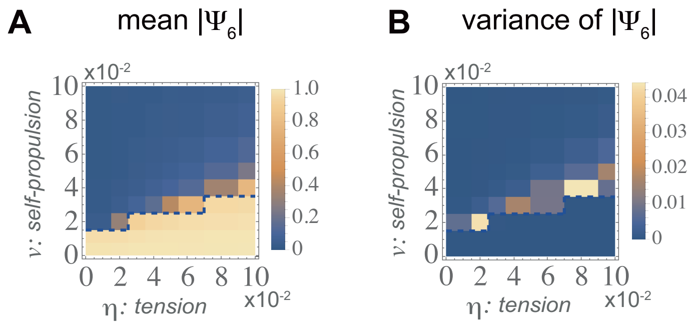

FIG. S4: The colormap of the hexatic order parameter. The order parameter $|\Psi_6|$ is defined by $|\Psi_6(\boldsymbol{r})| = \left|\sum_{j=1}^{n} e^{i6\theta_{ij}}/n\right|$. The mean (A) and variance (B) are plotted against v and η. The blue dashed lines represent the solid/fluid phase boundary judged by $D_{\rm eff}$ in the main text. High $|\Psi_6|$ values beyond the phase boundary can be attributed to the appearance of the hexatic phase (see also Figs. 4 a and b). The same parameters to fig. S2 are used.

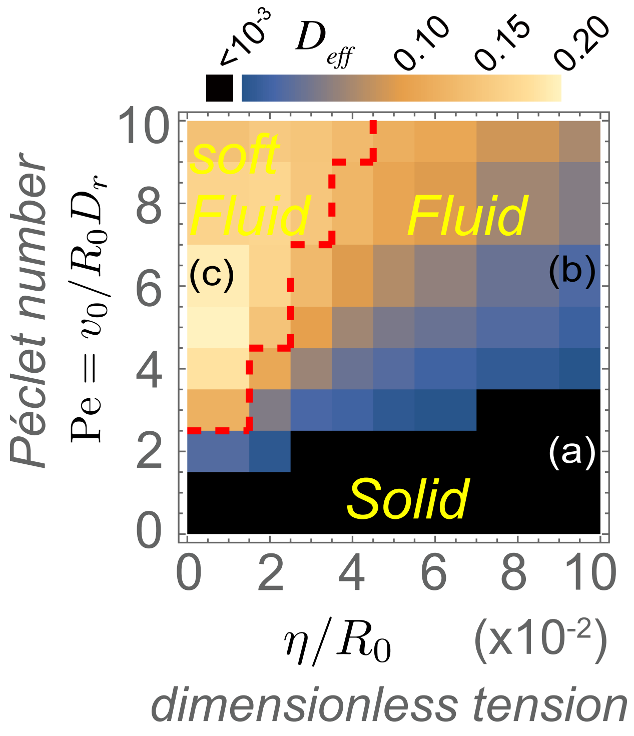

FIG. S5: Phase diagram based on dimensionless parameters. The same phase diagram to Fig. 2D using the Peclet number v_0/D_rR_0 and the dimensionless tension η/R_0. When a coefficient for the interaction Hamiltonian $\mathcal{H}_{int}$ is explicitly introduced as k, the non-dimensionalized Hamiltonian is given as $\mathcal{H}/kR_0^2 = \sum_{i<j}\int \phi_i\phi_j dxdy/R_0^2 + (\eta/R_0k)\sum_i \int_0^{2\pi}\sqrt{(R^i/R_0)^2 + (R^{i\prime}/R_0)^2}d\theta$. Here, η/R_0k is the dimensionless tension and takes the same value to η since $k = 1.0$ and $R_0 = 1.0$.

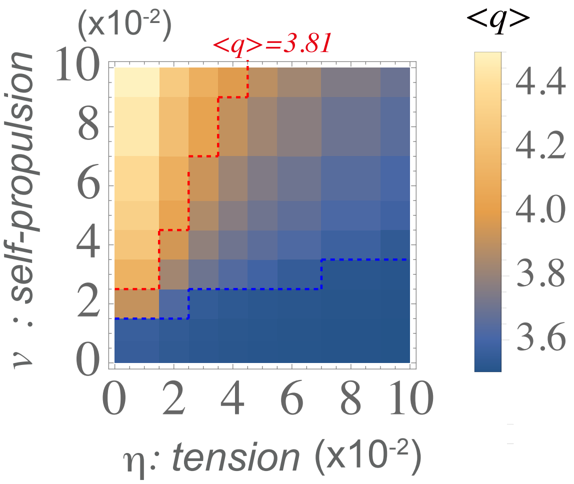

FIG. S6: The colormap for the mean value of the shape index. The red line displays $\langle q \rangle = 3.81$, whereas the blue dashed lines represent the solid/fluid phase boundary judged by $D_{\rm eff}$ in the main text. These calculations of $\langle q \rangle$ are based on the same simulation as Fig. 2 and figs. S2-4.

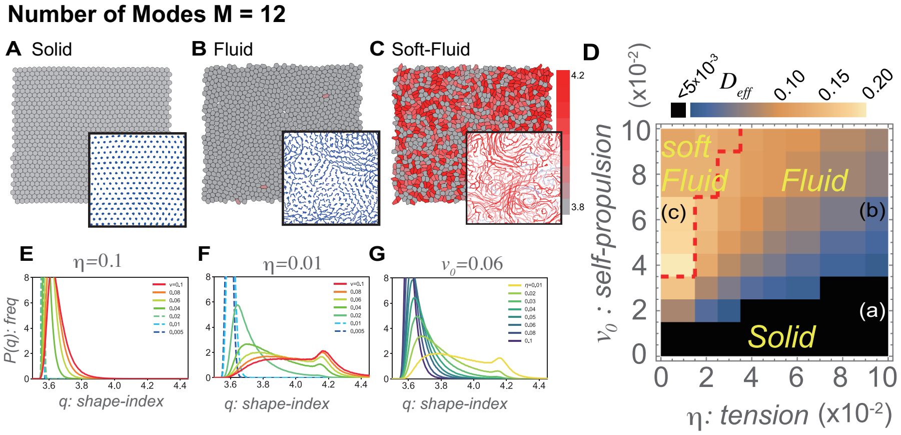

FIG. S7: Simulation results with up to 12th Fourier mode (M = 12). (A-C) The snapshot of the simulation for the solid phase (A), the fluid phase (B), and the soft fluid phase (C). The insets denote particle trajectories within 10^5 steps, and the color scale from blue to red represents the average of q during the time steps. (D) Phase diagram against the tension η vs. the self-propulsion velocity v_0. The color indicates the effective diffusion constant D_{eff} evaluated from simulation with $N = 1024$ and 1×10^7 time steps. The red dashed line represents $\langle q \rangle = 3.81$. (E-G) Distributions of the shape index for $\eta = 0.1$ (E) and $\eta = 0.01$ (F) with varying v_0, and that for $v_0 = 0.06$ with varying η (G). Thick lines indicate the parameters for the fluid or soft-fluid phase, and the dashed lines represent the parameters for the solid phase.

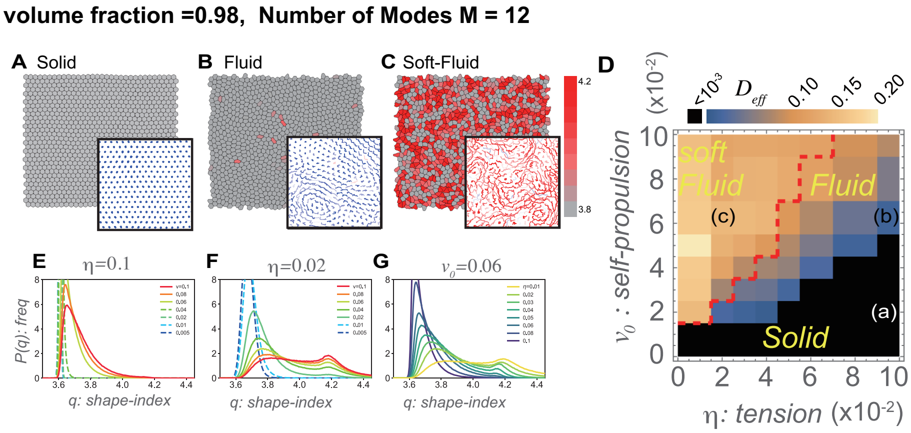

FIG. S8: Simulation results with packing fraction 0.98 and M = 12. (A-C) The snapshot of the simulation for the solid phase (A), the fluid phase (B), and the soft fluid phase (C). The insets denote particle trajectories within 10^5 steps, and the color scale from blue to red represents the average of q during the time steps. (D) Phase diagram against the tension η vs. the self-propulsion velocity v_0. The color indicates the effective diffusion constant D_{eff} evaluated from simulation with $N = 1024$ and 1×10^7 time steps. The red dashed line represents $\langle q \rangle = 3.81$. (E-G) Distributions of the shape index for $\eta = 0.1$ (E) and $\eta = 0.02$ (F) with varying v_0, and that for $v_0 = 0.06$ with varying η (G). Thick lines indicate the parameters for the fluid or soft-fluid phase, and the dashed lines represent the parameters for the solid phase.

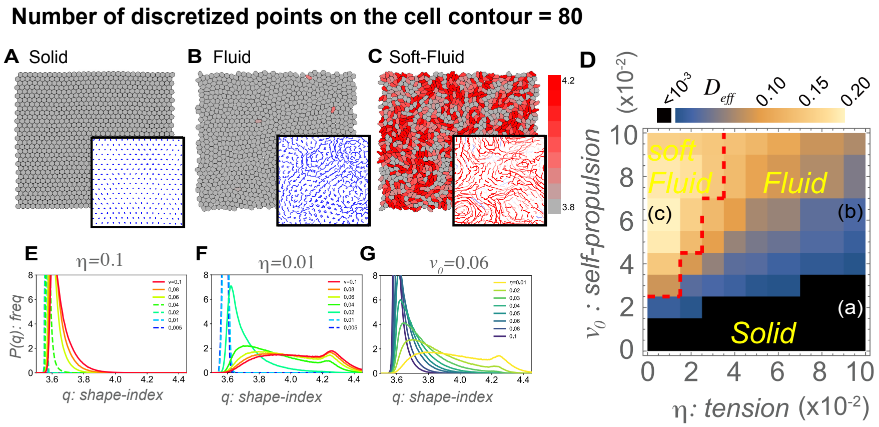

FIG. S9: Simulation results with 80 discretized points for the cell contour. (A-C) The snapshot of the simulations. (D) Phase diagram against the tension η vs. the self-propulsion velocity v_0. The color indicates the effective diffusion constant D_{eff} evaluated from simulation with $N = 1024$ and 1×10^7 time steps. The red dashed line represents $\langle q \rangle = 3.81$. (E-G) Distributions of the shape index for $\eta = 0.1$ (E) and $\eta = 0.01$ (F) with varying v_0, and that for $v_0 = 0.06$ with varying η (G).

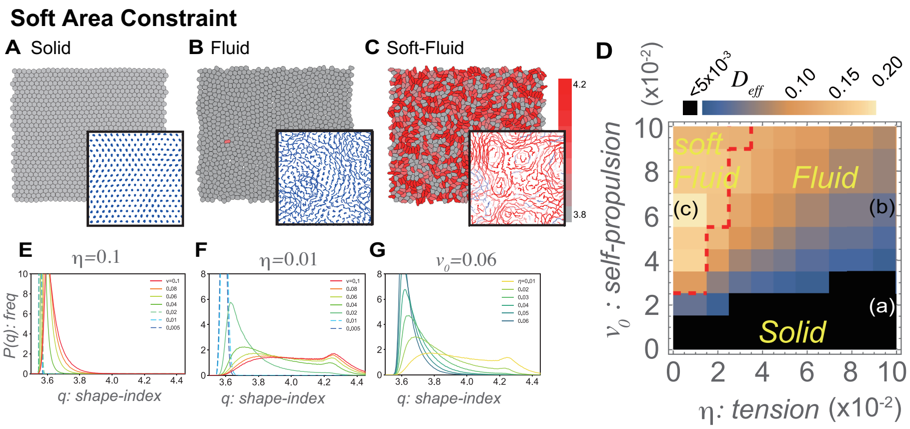

FIG. S10: Simulation results with a soft-constraint of the area conservation. The penalty parameter was set to $M_A = 50$. The details of the model are given in the Supplemental Text. (A-C) The snapshot of the simulation for the solid phase (A), the fluid phase (B), and the soft fluid phase (C). The insets denote particle trajectories within 10^5 steps, and the color scale from blue to red represents the average of q during the time steps. (D) Phase diagram against the tension η vs. the self-propulsion velocity v_0. The color indicates the effective diffusion constant D_{eff} evaluated from simulation with $N = 1024$ and 1×10^7 time steps. The red dashed line represents $\langle q \rangle = 3.81$. (E-G) Distributions of the shape index for $\eta = 0.1$ (E) and $\eta = 0.01$ (F) with varying v_0, and that for $v_0 = 0.06$ with varying η (G). Thick lines indicate the parameters for the fluid or soft-fluid phase, and the dashed lines represent the parameters for the solid phase.

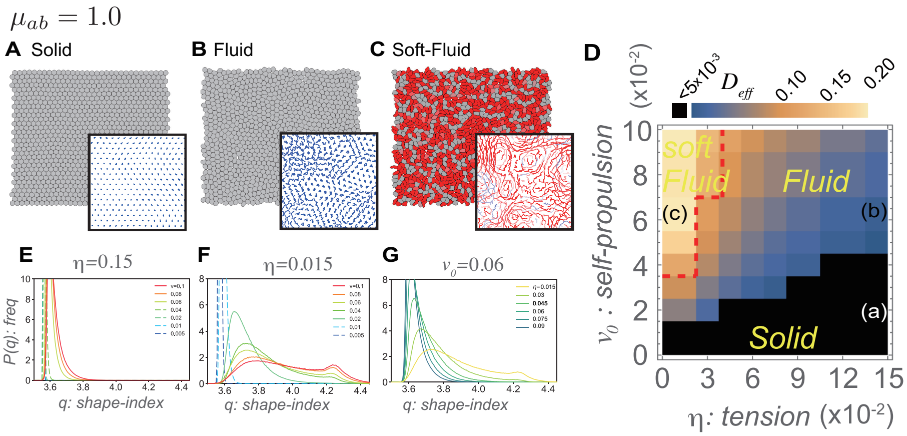

FIG. S11: Simulation results with a higher value of the mobility parameter for a_n and b_n ($\mu_{ab} = 1.0$). (A-C) The snapshot of the simulation. (D) Phase diagram against the tension η vs. the self-propulsion velocity v_0. The color indicates the effective diffusion constant D_{eff} evaluated from simulation with $N = 1024$ and 1×10^7 time steps. Note that the tension parameter η takes a different range $\eta = 0.015 - 0.15$ from the original range ($\eta = 0.01 - 0.1$) in Fig. 3 in the main text. (E-G) Distributions of the shape index for $\eta = 0.1$ (E) and $\eta = 0.01$ (F) with varying v_0, and that for $v_0 = 0.06$ with varying η (G). Thick lines indicate the parameters for the fluid or soft-fluid phase, and the dashed lines represent the parameters for the solid phase.

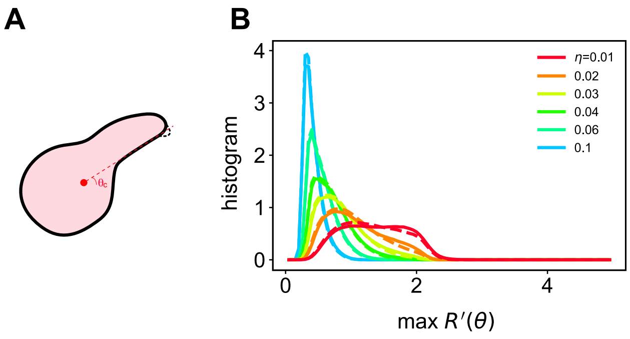

FIG. S12: Distributions in $\max|R'|$ **for both fluid phases with** $v = 0.06$**.** (A) The schematic illustration at the vicinity of the breakdown of the single-value assumption for $R^i(\theta)$. The red circle denotes the cell center, and $R^i(\theta)$ shows a large jump at θ_c. (B) The distribution of $\max_\theta |dR^i(\theta)/d\theta|$ for v=0.06 and N=1024. Thick and dashed lines indicate results with the number of discretized points on the cell contour =40 and 80. For the soft-fluid phase with $\eta = 0.01$ and 0.02, this value takes a relatively larger value (≈ 2.0) compared with that in the fluid phase with $\eta = 0.03 - 0.1$, however, it is much smaller than its upper bound ($\max|R'| \approx 5.0$). Here, the upper bound can be calculated from the constraint $\sum_{n=2}\sqrt{a_n^2 + b_n^2} < \sqrt{2/3}$. Furthermore, $\max|R'| \approx 2.0$ means that the largest change in $R^i(\theta)$ with respect to the change $\theta \to \theta + d\theta$ ($d\theta = 2\pi/80$) is around $R_0/6$ which is much smaller than the cell radius R_0. These estimates imply that the single-value assumption still holds in the soft-fluid phase.

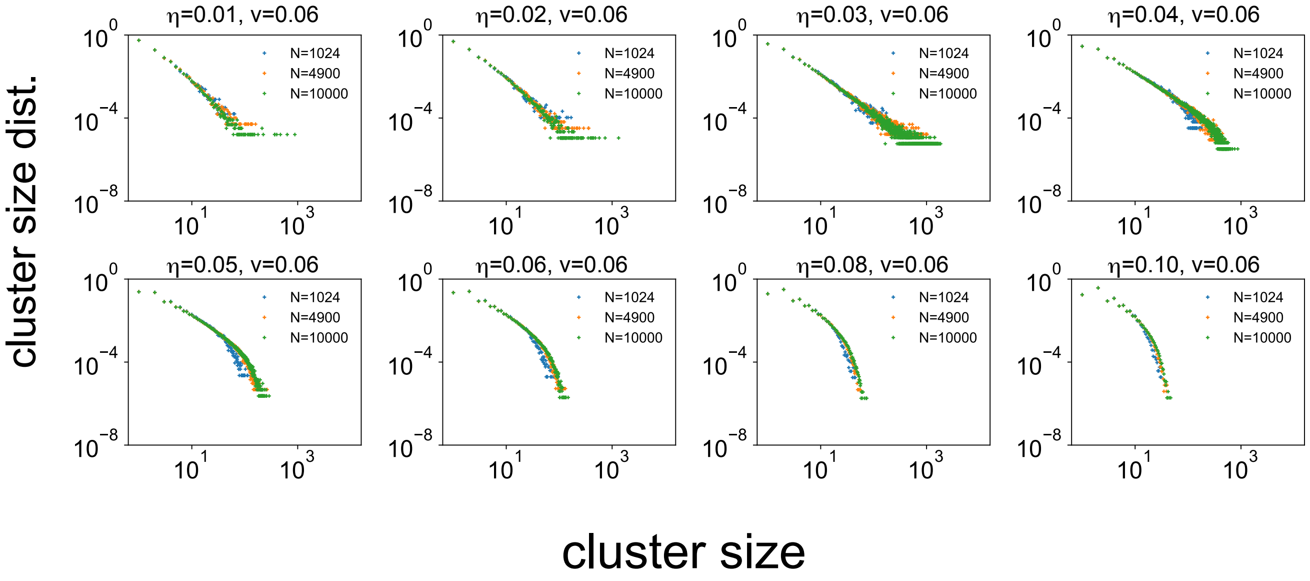

FIG. S13: The size distribution of the cluster of the topological defects for $N = 1024, 4900$ **and** 10000 **with the volume density 0.95.** The panel with $\eta = 0.03$ and $v_0 = 0.06$ shows the same figure as Fig. 3(c).

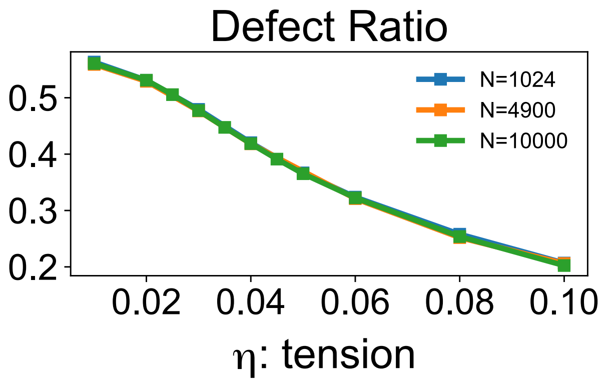

FIG. S14: The ratio of the topological defects. The defect ratio does not show a significant change by changes in N.

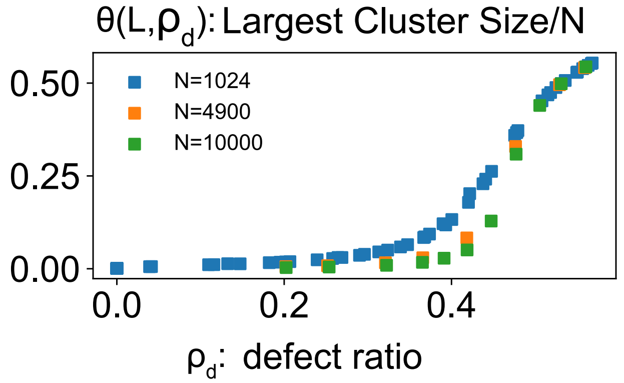

FIG. S15: Scatter plot for the largest cluster size of the topological defects against the defect ratio ρ_d. The time average of the largest cluster size divided by N, $\theta(L, \rho_d)/N$, is plotted against the mean of the defect ratio ρ_d. This calculation is based on simulation with different N, v_0, and η. See also Fig. 3d for the plot with the finite size scaling.

SUPPLEMENTAL MOVIE1: A time series of the simulation with 10^4 particles with volume density 0.8. The same simulation to Fig.1(c). The total simulation time steps are 5×10^6. The red color represents the shape-index ($perimeter/\sqrt{area}$) for each particle.

SUPPLEMENTAL MOVIE2: A time series of the simulation with 1024 particles with volume density 0.95. The same simulation to Fig.2(a) ($\eta = 0.1$ and $v_0 = 0.02$). The red color represents the shape-index (perimeter area) for each particle.

SUPPLEMENTAL MOVIE3: A time series of the simulation with 1024 particles with volume density 0.95. The same simulation to Fig.2(b) ($\eta = 0.1$ and $v_0 = 0.06$). The red color represents the shape-index (perimeter area) for each particle.

SUPPLEMENTAL MOVIE4: A time series of the simulation with 1024 particles with volume density 0.95. The same simulation to Fig.2(c) ($\eta = 0.01$ and $v_0 = 0.06$). The red color represents the shape-index (perimeter area) for each particle.
